# Whole Genome Mapping with Feature Sets from High-Throughput Sequencing Data

**DOI:** 10.1371/journal.pone.0161583

**Published:** 2016-09-09

**Authors:** Yonglong Pan, Xiaoming Wang, Lin Liu, Hao Wang, Meizhong Luo

**Affiliations:** National Key Laboratory of Crop Genetic Improvement and College of Life Science and Technology, Huazhong Agricultural University, Wuhan, 430070, China; Clemson University, UNITED STATES

## Abstract

A good physical map is essential to guide sequence assembly in *de novo* whole genome sequencing, especially when sequences are produced by high-throughput sequencing such as next-generation-sequencing (NGS) technology. We here present a novel method, Feature sets-based Genome Mapping (FGM). With FGM, physical map and draft whole genome sequences can be generated, anchored and integrated using the same data set of NGS sequences, independent of restriction digestion. Method model was created and parameters were inspected by simulations using the Arabidopsis genome sequence. In the simulations, when ~4.8X genome BAC library including 4,096 clones was used to sequence the whole genome, ~90% of clones were successfully connected to physical contigs, and 91.58% of genome sequences were mapped and connected to chromosomes. This method was experimentally verified using the existing physical map and genome sequence of rice. Of 4,064 clones covering 115 Mb sequence selected from ~3 tiles of 3 chromosomes of a rice draft physical map, 3,364 clones were reconstructed into physical contigs and 98 Mb sequences were integrated into the 3 chromosomes. The physical map-integrated draft genome sequences can provide permanent frameworks for eventually obtaining high-quality reference sequences by targeted sequencing, gap filling and combining other sequences.

## Introduction

Since 2005, the number of registered genome sequencing projects has doubled every two years, reaching 11,472 as of September, 2011 [1]. Recent projects have expended a tremendous amount of effort to sequence more complex genomes [2]. Many projects aimed to generate reference genome sequences for the genus or species of interest. A reference genome sequence is an important tool to explore genome structure and function, identify genomic variations, infer information about species evolution, and guide the genome assembly of closely related species [3–8]. However, in all cases, the high quality of a reference genome sequence is critical to ensure reliable outcomes [9].

Two approaches, clone-by-clone (CBC) and whole genome shotgun (WGS), were developed for whole genome sequencing [10–13]. WGS has been widely used along with high-throughput sequencing such as next-generation sequencing (NGS) technologies [14]. Due to the high-throughput and cost-effective nature, many genomes have been sequenced using WGS/NGS. However, this approach suffers from the key problem that the NGS reads are too short to reliably locate and order scaffolds on chromosomes and complete chromosome assemblies, especially when a genome is large and contains an abundance of repetitive sequences, large gene families, and extensive segmental duplications [5]. As the development of the single-molecule sequencing or the third generation sequencing technology, longer sequencing reads and more continuous contigs could be obtained [15–17]. However, the technology alone is still difficult to complete sequences of complex genomes at the present. CBC does not suffer from these problems and is considered a “gold standard” for genome sequencing [18, 19]. In the CBC approach, a physical map is first constructed using large-insert clones, mainly bacterial artificial chromosomes (BACs) [20] and used as a framework for the allocation of assembled sequences to chromosomes [10, 12, 21]. Physical clone maps are also important tools for locating genes for map-based cloning [22, 23], assembling genomic repeats [24] and filling gaps [25].

Fingerprinting technology has been widely used for physical clone mapping [26–28]. In this technology, large insert clones such as BACs are fingerprinted with restriction enzyme(s), and the shared restriction bands are used to identify overlaps between clones [29]. This technology has been implemented in automated and high-throughput systems [26]. However, it is costly and has a limited resolution for large genome mapping [30]. Optical mapping [31], nanochannel genome mapping [32] and whole genome profiling (WGP) [30] methods have been developed as alternatives to construct BAC physical maps. But they, as well as fingerprinting, were all designed specifically for physical mapping only and are based on restriction digestion. Uneven distributions of restriction sites throughout genomes, and possible ineffective or incomplete digestion would thus influence the outcomes.

Previously, to integrate physical map with sequence map [33] or gene map [34], separate data sets or projects for physical mapping and sequencing were needed. Here we present a novel method, Feature sets-based Genome Mapping (FGM). With FGM, physical clone map-integrated draft whole genome sequence can be generated, assembled and anchored using the same set of NGS sequences, independent of restriction digestion. This method has tremendous advantages over other existing methods and is expected to be used to construct *de novo* reference sequences for a broad range of species.

## Results

To execute the workflow, a model concerning all steps was established. Many model parameters or conditions that would affect the result were considered. First, to find and optimize the most critical parameters, many simulations were processed *in silico*. In these simulations, it was critical to keep true feature sequences (FSs) and k-mers and remove false ones when resolving the feature sequence set (FS-sets) and the k-mer set (K-sets) of each clone. Then, a genome sequence from a simulation with a given critical parameter combination was obtained, and the original and simulated assembled genome sequences were compared to verify the effectiveness of the method. Finally, an experiment was performed with the critical parameter combination to test the difference between the theoretical and experimental results.

### Workflow of Feature Sets-based Genome Mapping

**Fig 1:**
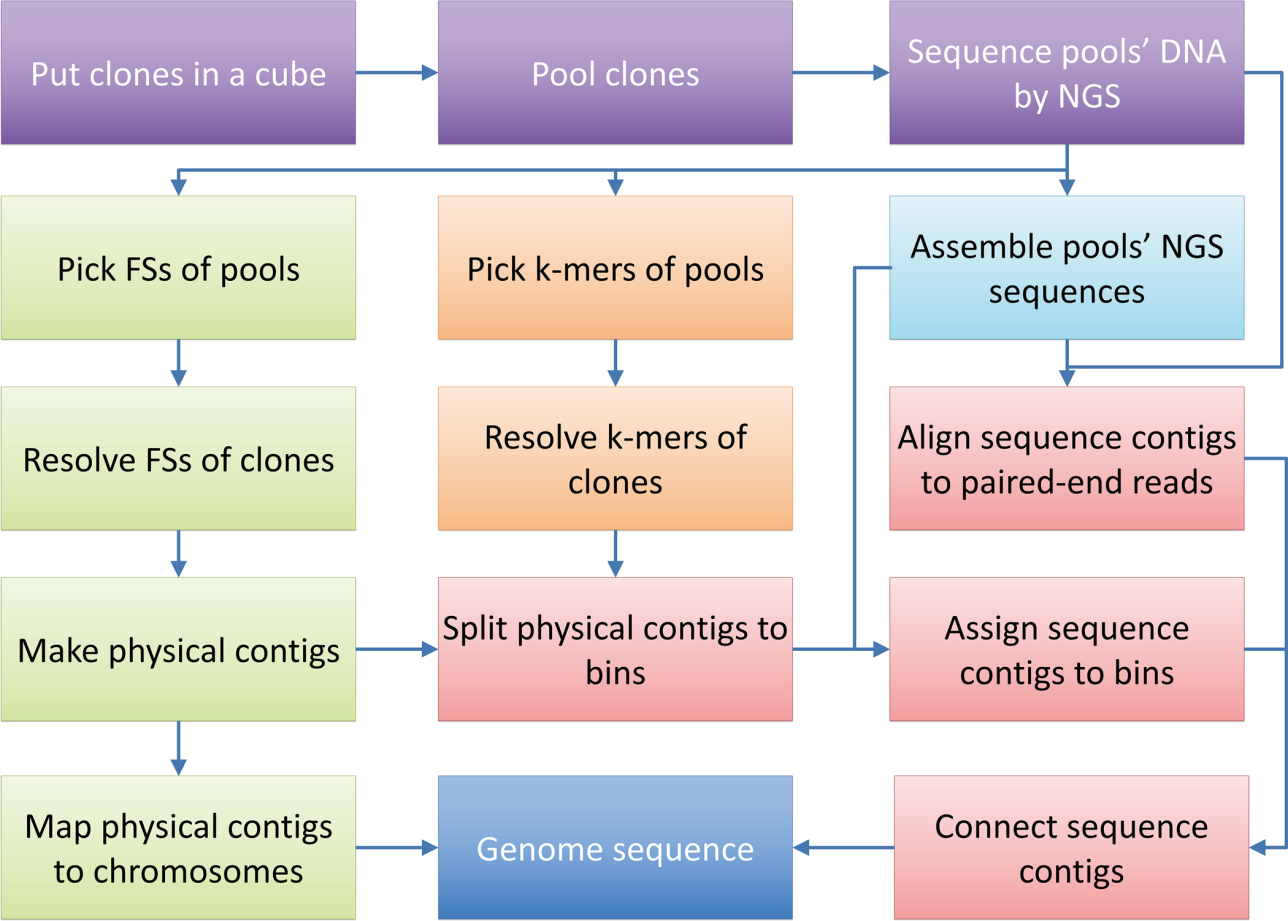
The workflow of the method consisting of four main steps. 1) Pick clones and combine them to pools, and sequence the pools’ DNA by NGS; 2) Resolve each clone’s F-sets; 3) Make a physical map with the clones’ FS-sets and split physical contigs into bins according to the clones’ overlap and K-sets; 4) Assemble pools’ NGS reads into sequence contigs, allocate sequence contigs to the physical map, and connect the allocated sequences to form longer sequence scaffolds.

The workflow of feature sets-based genome mapping consists of four main parts (Fig 1):

1. Constructing pools and sequencing pool DNA by NGS
  Pools containing multiple clones were formed following the rule that each clone was located by more than 3 pools. DNAs of all pools were extracted and sequenced by NGS. All sequence reads of each pool were assembled using a short-read assembler to obtain the sequence contigs.
2. Resolving the F-sets (Feature sequence sets and K-mer sets) of clones
  All sequence reads from NGS of pools were screened. The upstream sequences of a given length (31 bp in this paper) of selected prefix sequence(s) were selected as FSs (S1 Algorithms Section A). Through intersecting Feature sequence sets of pools, clones’ intersected FS-sets could be obtained. Most errors in each intersected FS-set were removed at the refining step to obtain the final FS-set (S1 Algorithms Section C). Similarly, all sequence reads of each pool were screened again to find K-mer sets. For a given pool or clone, its FS-set is a subset of its K-set. The final K-sets of all clones were obtained using the same algorithms.
3. Contiging the physical clone map
  The FS-sets of clones were converted to “.size” files compatible with the program of FingerPrinted Contig (FPC, v9.4) [35], and contiging was performed to accomplish the physical mapping. According to the overlap of clones on physical contigs, the physical contigs were split into bins.
4. Integrating the sequence contigs to the physical map
  Paired-end alignments between NGS sequence reads of pools and sequence contigs were executed using Bowtie 2 [36]. Sequence contigs assigned to proximal bins were connected to sequences of physical contigs. If enough markers were present on clones, the sequences of physical contigs could be mapped and connected to chromosomes.

### Simulations

Parameters such as sequencing depth, read length, pooling dimensions, sequencing errors and pool coverage are clearly the very important factors controlling the outcomes, and interact with each other. Therefore, simulations were performed focusing on these parameters.

#### Preparation of simulation data

The genome sequences in all simulations to determine the parameters were from the *Arabidopsis thaliana* Columbia sequence. The BAC library was generated *in silico* using parameters based on a BAC library of maize constructed in our laboratory [37]. A total of 10,000 BAC clones were used to analyze the frequency distribution of insert sizes. Insert sizes ranged between 60 and 300 kb; the average clone insert size was 137.42 kb and the variance was 417.54 kb^2^, in agreement with the designed distribution (S1 Results Section A). Sequence quality was also imported to the simulations by a quality matrix from more than 100 billion sequence reads of Illumina/Solexa Genome Analyzer (S1 Table). The average error probabilities at each base site of reads were calculated based on the quality matrix. The error probability ranged from a minimum of 0.10% to a maximum of 16.91%, increasing as the read length increased. The average error probability of all reads was 2.46%. According to the quality matrix, approximately 456 million reads were generated and analyzed; the normalized quality distribution agreed with the expected quality matrix defined above (S1 Results Section B).

In subsequent simulations, DNAs of all pools were paired-end sequenced within this quality matrix, and each read was 100 bp in length.

#### Parameters of sequencing depth, pooling strategy and sequence errors

A suitable sequence depth is necessary because of the read length limitation and the possibility of sequencing errors. During these simulations, BAC library coverage less than 1X was used to decrease the influence of overlap between clones. The k-mer length and FS length were set to 31, and prefix sequences were “GGATCC” and “GAATTC”.

**Fig 2:**
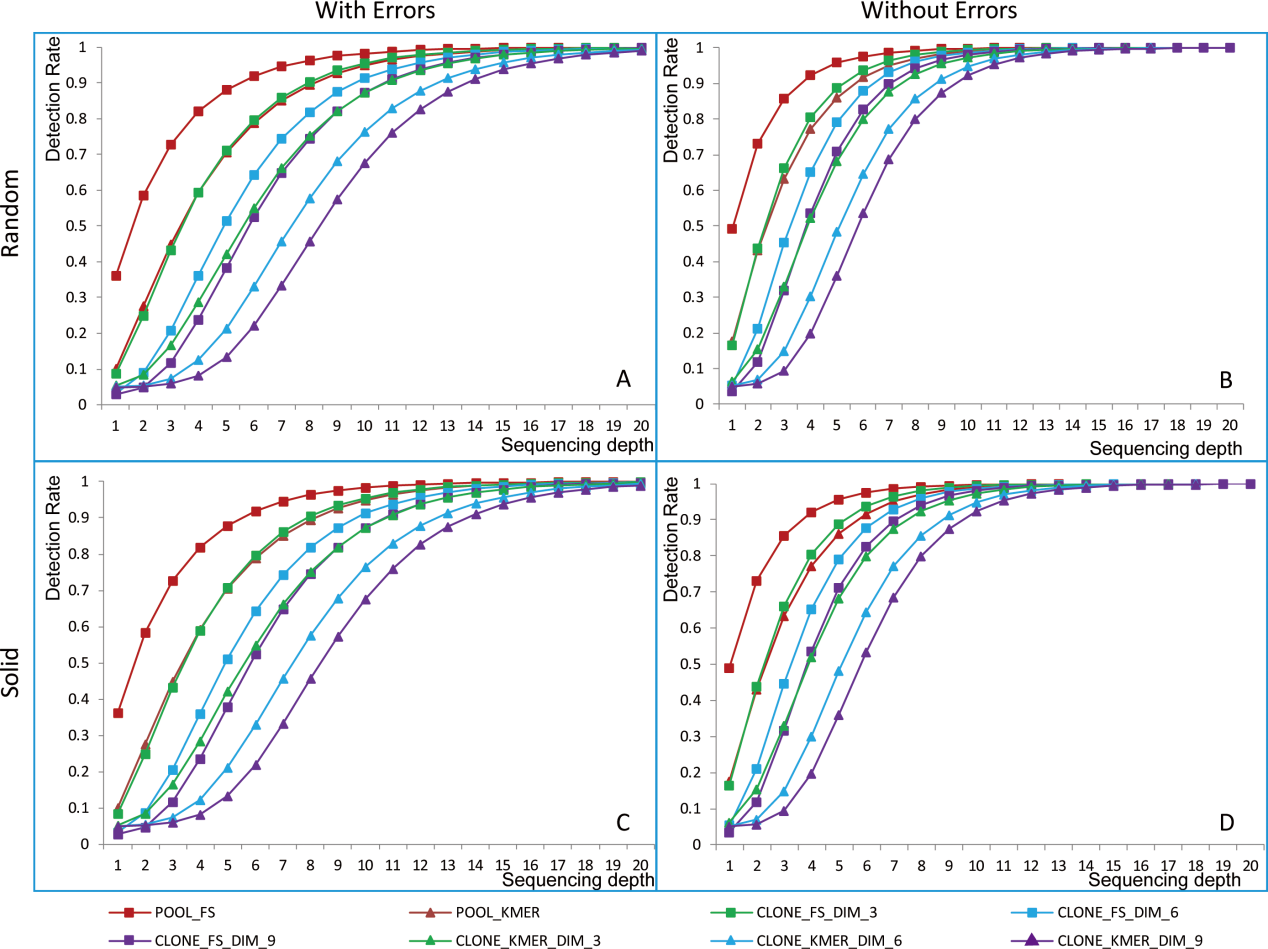
Detection rates of F-sets. The abscissa axis represents sequencing depth, and the vertical axis represents detection rate. “FS” means feature sequence, “KMER” means k-mer, and “DIM” means the pools’ dimensions. (A) and (B) were simulated using a random pooling strategy. (C) and (D) were simulated using a solid pooling strategy. (A) and (C) were simulated with sequencing errors. (B) and (D) were simulated without sequencing errors. Curves with squares indicate the trend of the detection rate of FS. Curves with triangles represent the trend of the detection rate of k-mer. Red curves represent pools. Green, blue and purple curves represent clones in 3D, 6D and 9D pooling, respectively.

F-sets’ detection rates of pools and clones were analyzed for different sequencing depths, pooling strategies, pool dimensions (defined in the S1 Material and Methods Section A List 2) and sequencing errors (Fig 2). Comparing random (Fig 2A and 2B) and solid pooling strategies (Fig 2C and 2D) (defined in the S1 Material and Methods Section A List 2), the curves of detection rates of F-sets of clones are similar. Comparing the results with sequencing errors (Fig 2A and 2C) and without (Fig 2B and 2D), sequencing errors have an enormous effect on the detection of F-sets. For a critical detection rate value of 0.98, sequencing depths of 11X, 12X and 13X are enough for the pool dimensions of 3D, 6D and 9D without sequencing errors, but 16X, 18X and 19X are necessary with sequencing errors (S2 Table). In terms of probability, larger pool dimensions reduce the number of false positives and decrease the detection rate. To guarantee the detection of enough elements in F-sets, a sequencing depth of 19X is sufficient for a critical detection rate of 0.98 for 9D pooling.

In subsequent simulations, we set the parameter of sequencing coverage to 20X in solid pooling strategy with sequencing errors.

#### Parameters of filtering frequency, pool coverage and pooling dimension

Pools’ F-sets were filtered by element frequency to remove most false elements before intersecting the pools’ F-sets. A statistical analysis of the frequency in the screened pools’ F-sets was performed, indicating that most false elements imported by sequencing errors occurred only one time (S3 Table). Then the elements that occurred only one time (the filtering frequency equals 1) in pools’ F-sets were removed.

When combining clones into pools, the pool coverage, the ratio of total insert size of all clones in the given pool to genome size, should be limited. Greater pool coverage results in more false elements in clones’ intersected F-sets and the retention of fewer true elements (FSs or k-mers) in final F-sets. To obtain the best possible pool coverage, we inspected pool coverage at different discrete values with 3 pooling dimensions.

**Fig 3:**
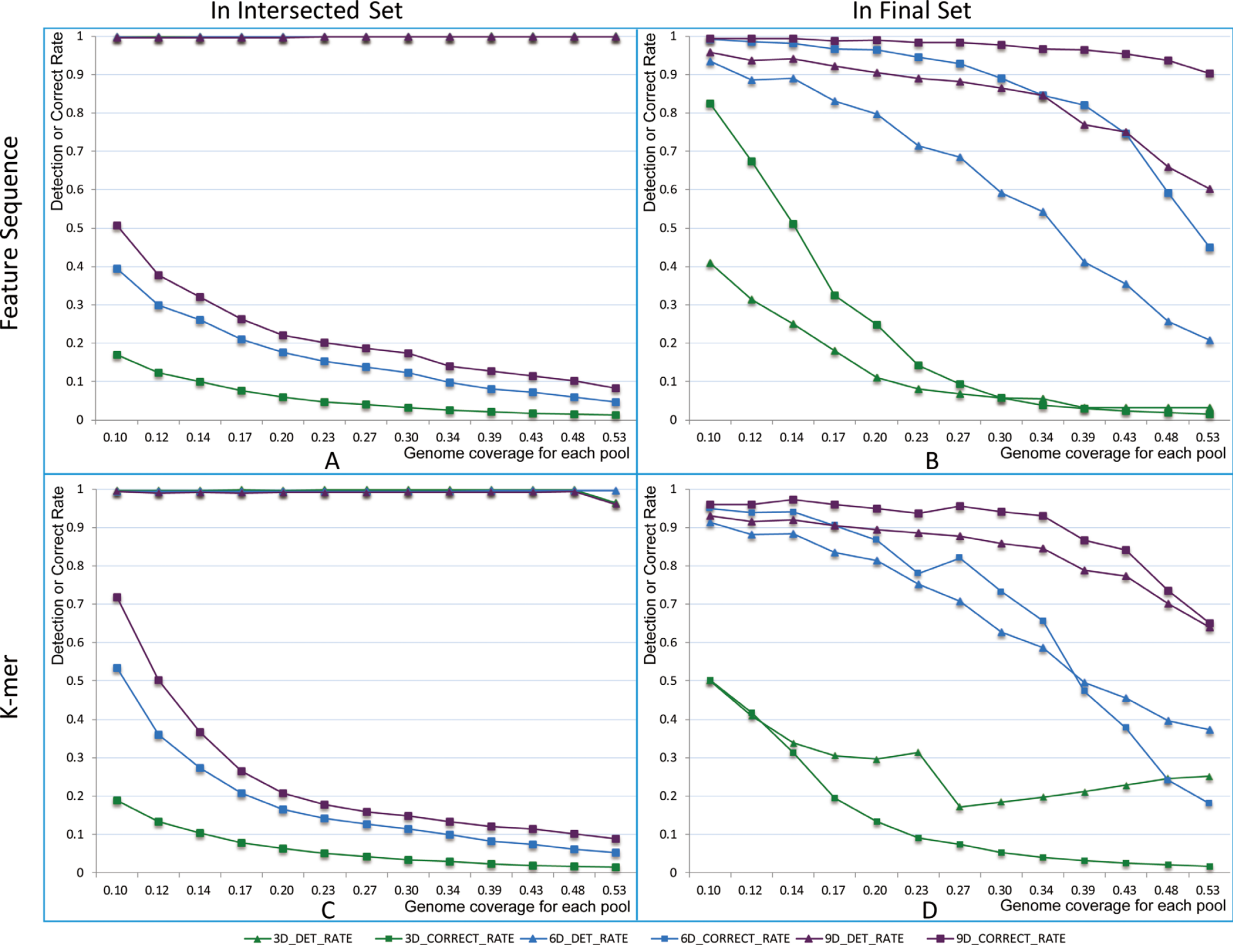
Detection and correct rates of the intersected and final F-set. The abscissa axis represents pool coverage, and the vertical axis represents detection rate or correct rate. (A) and (B) were simulated for FS. (C) and (D) were simulated for k-mer. (A) and (C) were statistics from clones’ intersected F-sets. (B) and (D) were statistics from clones’ final F-sets. Curves with squares show the trend of detection rate. Curves with triangles show the trend of correct rate. Green, blue and purple curves represent clones in 3D, 6D and 9D pooling, respectively.

A new concept, the correct rate of the clone’s F-set, is defined as the ratio of the true element number in resolved F-sets to the size of real sets. More than 99.6% of elements were detected in clones’ intersected F-sets for all pool dimensions (Fig 3A and 3C) under the sequencing conditions given above. However, many elements were false, as reflected by the correct rate. The correct rate of clones’ intersected F-sets was lower with greater pool coverage, especially for 3D pooling (pools’ demission is 3D, S1 Material and Methods Section A List 2). Too many false elements were present in intersected F-sets to contig clones and locate sequences. Refining step removed most false elements while most true elements were retained in the final F-sets if the pool coverage for 6D and 9D pooling was limited (Fig 3B and 3D). Overall, the correct rate decreases with increasing pool coverage. For 3D pooling, the detection rate was less than 20%, and the correct rate was less than 10% when the pool coverage was greater than 0.27. For 6D pooling, the correct rates are 89.01% and 73.02% for the final FS-set and the final K-set, respectively, when the pool coverage is 0.30. Compared to 3D and 6D pooling, the correct rate in the clones’ F-sets is much higher for 9D pooling, decreasing more slowly with increasing pool coverage.

**Fig 4:**
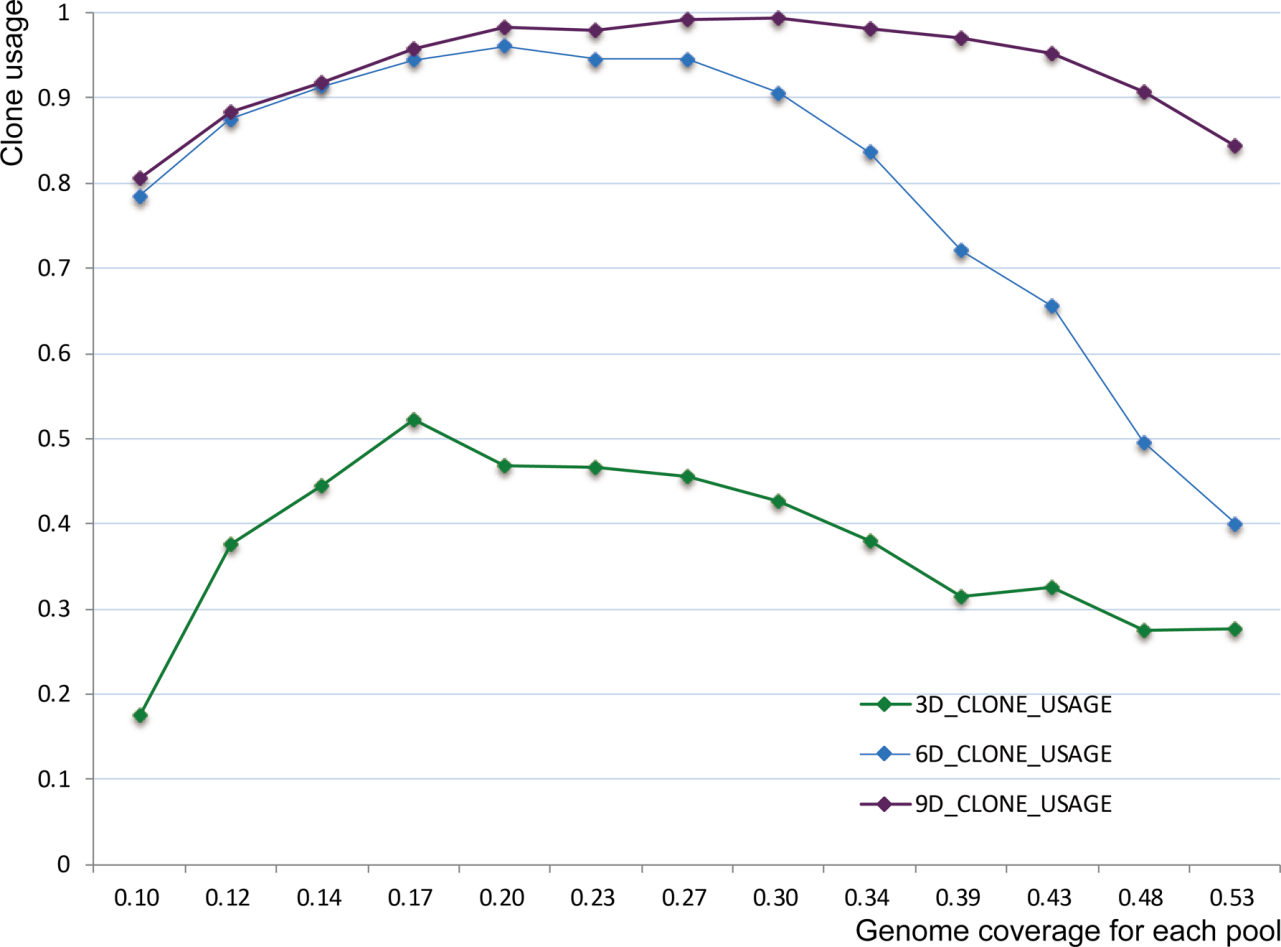
Clone usage at different levels of pool coverage. The abscissa axis represents pool coverage, and the vertical axis represents clone usage.

All FSs in all final FS-sets were transformed and used to build physical map by FPC program [35]. Then a statistical analysis of clone usage was carried out to determine the effect of pool coverage (Fig 4). Clone usage is the ratio of the number of clones in contigs to all clones imported to FPC. Each curve on the statistics of clone usage exhibited a rise and fall and included a maximum value; the maximum values were 52.20%, 96.09% and 99.39% at 0.17, 0.20 and 0.30 for 3D, 6D and 9D pooling, respectively. Therefore, we designated 90% as the critical value of clone usage, and subsequent simulations were performed at this value. The critical value of pool coverage should be 0.30 for 6D pooling and 0.48 for 9D pooling (S4 Table).

#### Sequence assembly, integration and validation

After the most important parameters were inspected and determined, the procedure of whole genome assembly in simulation were performed to validate the critical parameters. Briefly, 4096 BAC clones were generated and assigned to 6D pool in solid pooling strategy, and each pool contained 256 clones; DNAs of all pools were paired-end sequenced within the quality matrix, and each read was 100 bp in length; the sequence coverage of each pool was 20X. All steps were performed as previous simulations.

Total 807,584 feature sequences were indexed to 97,492 types, and each clone contained 197 indexes in average. Then, the feature sequence indexes of all clones were imported into the program of FPC. A physical map was constructed at the cutoff of 10^−12^, tolerance of 0 and other default parameters. After DQ analyzing at the step of 9, problematic contigs were split to remove the Q-clones until no contigs contained more than 5 Q-clones (http://www.agcol.arizona.edu/software/fpc/FPChelpdoc.htm). Total 4021 clones were assembled to 220 contigs, which indicated the clone usage was 98.16%.

**Fig 5:**
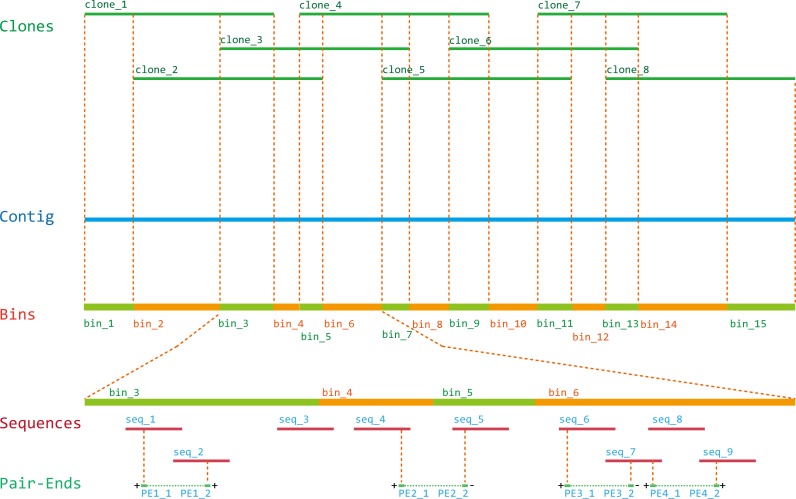
Splitting a contig into bins according to the clones’ order in the physical contig. The k-mer set of each bin was derived from the intersections or differences among overlapping clones. Assembled sequences were allocated to the best bins. Paired-end sequences were used to connect assembled sequences located at the same or nearby bins to form larger sequences. The orientation of connected sequences was determined by the sequence loci and the directions of paired-end alignments. The red blocks labeled with the prefix “seq” indicate assembled sequences from the long-read assembler. The prefix “PE” labels the paired-ends. The symbol “+” or “-” indicates the directions of paired-end alignments.

Approximately 108.2 Mb sequences were obtained after reassembly by the long-read assembler. Sequences shorter than 100 bp were filtered, and then the retained sequences were allocated to bins. In total, 103.1 Mb sequences with an N50 size of 37.91 Kb were allocated and the longest sequence was 176.18 Kb. Using the integrating strategy (Fig 5 and S1 Material and Methods Section A List 6), the K-sets of clones were split to connect sequences of physical contigs. Total 109.9 Mb sequences of physical contigs with an N50 size of 663.31 Kb were connected by paired-end alignments, meaning that approximately 91.58% of the Arabidopsis genome sequence (about 120 Mb) was mapped. Of the connected physical contigs, the longest one was 2.50 Mb. The physical contigs were reoriented and assigned to chromosomes by 1,515 markers. After filling 50 Kb of “N”s into the gaps between sequences of physical contigs mapped on chromosomes, a total of 118.4 Mb sequences for 5 Arabidopsis chromosomes were obtained.

**Fig 6:**
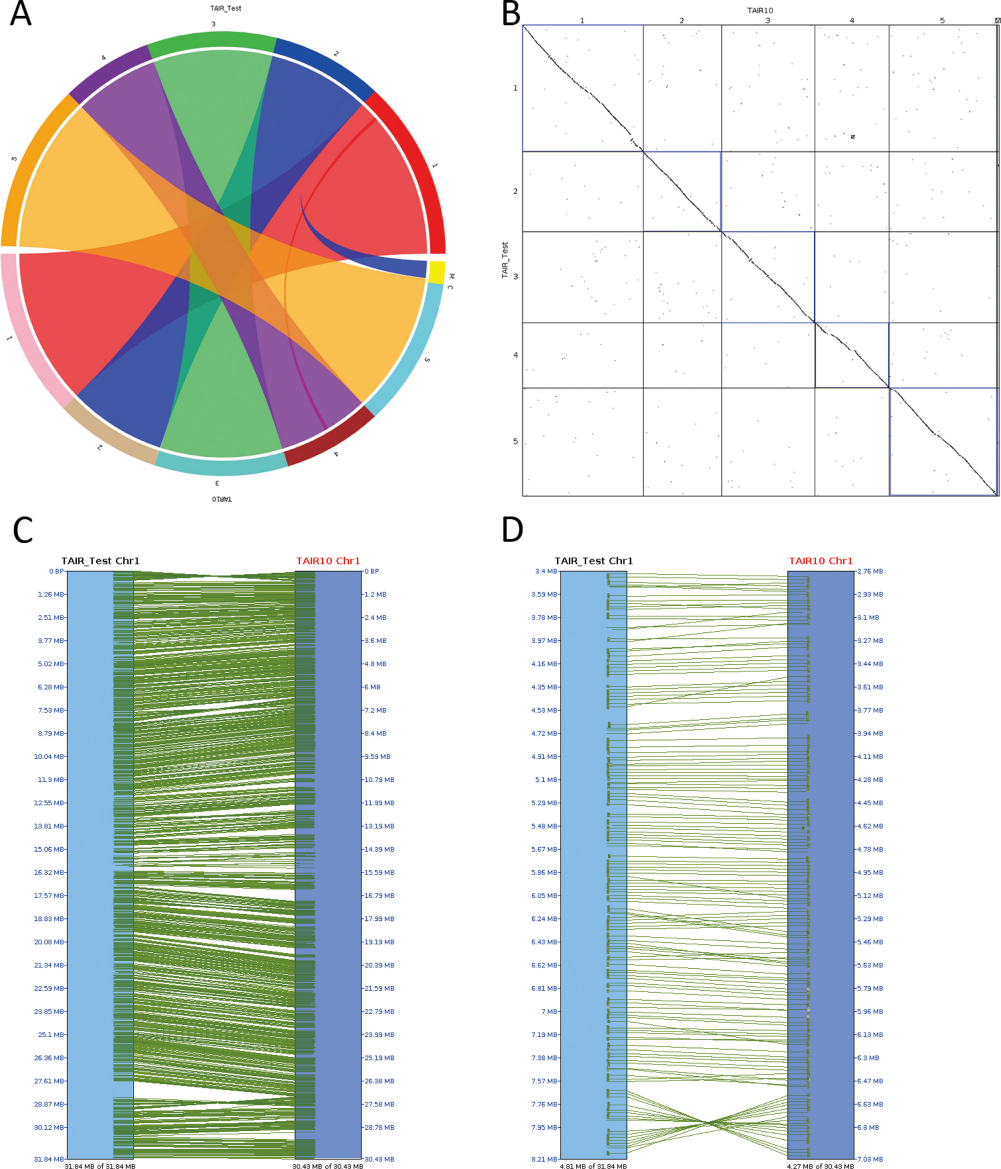
Comparison between the original and simulated genome sequences. “TAIR_Test” represents the simulated sequence and “TAIR10” represents the original genome sequence. “M” means mitochondria and “C” means chloroplast. (A) Circle view of the alignments. The upper semi-circle displays the chromosomes of “TAIR-Test” and the lower semi-circle displays the chromosomes of “TAIR10”. (B) Dot-plot view of all chromosomes. (C) Full view of the alignments of chromosome 1. (D) Segment detail of the alignments of chromosome 1.

To validate the assembled genome sequences, the original and assembled chromosomes (Fig 6, S1 Results Section C) were compared with the SyMAP program [38, 39]. As shown by the circle view of all chromosomes (Fig 6A), most sequences were mapped to the correct locations. Only one physical contig of chromosome 4 was mapped in error to chromosome 1. The chloroplast and mitochondrial sequences were not mapped because no molecular markers existed on those clones. The full alignments between all chromosomes are shown in a dot-plot view (Fig 6B). Some inverted segments were present in simulated sequences. For example, assembled chromosome 1 contained 3 large inverted segments (Fig 6C) because each related physical contig contained only one molecular marker. Some smaller segments could be mis-ordered and/or mis-oriented (Fig 6D) if the related sequence contigs were assigned to single bins on physical contigs without paired-end matches in close bins.

### Experimental validation

The simulations provided a guide to parameter combination. An experiment was performed to verify the validity of the model. A total of 4096 BAC clones with an average insert size of 113 kb were selected from the rice 93–11 BAC physical map [9], of which 4064 clones were from approximately 3 tiles of chromosome 1, 2 and 4, and 32 clones were from other chromosomes. Then, 6D pools were constructed with each dimension containing 16 pools, and with each pool containing 256 clones (S1 Data). The pool coverage was estimated to be approximately 0.33 (for a total chromosome length of 115 Mb), but the actual pool coverage could be greater than this estimate. The plasmid DNA of each pool was paired-end sequenced by Illumina/Solexa Genome Analyzer. The read length was 100 bp, and the distance between paired-end sequences was approximately 500 bp. The sequencing depth of each pool was approximately 30X. Approximately 20X coverage of relatively high-quality reads were used to screen F-sets of pools.

A total of 807,584 feature sequences were indexed to 191,117 types, and each clone contained 107 indexes in average. Then, the clones less than 40 indexes were ignored and the indexes of the retained 3558 clones were imported into the program of FPC. As the method developed in the above simulations, a physical map was constructed at the cutoff of 10^−20^, tolerance of 0 and other default parameters. A DQ analyzing at the step of 9 was performed and the problematic contigs were removed until no contigs contained more than 5 Q-clones. The physical map contained 452 contigs and 3,364 clones, indicating a clone usage of 82.12% (S1 Results Section D). The clone usage is slightly lower than expected (83.59% was expected at pool coverage of 0.34). In comparison, the fingerprinting data [9] of these clones were retrieved to construct the physical map at the cutoff of 10^−20^, tolerance of 7 and other default parameters. A DQ analysis was not performed because all contigs contained less than 5 clones. Only 845 clones were connected to 354 contigs; the others were all singletons.

Because no molecular markers were available on the rice 93–11 clones, 317 physical contigs were allocated to the chromosomes of *Oryza sativa ssp*. *japonica* Nipponbare (Build 5; http://rgp.dna.affrc.go.jp) by the available BAC end sequences (BESs) from our previous work [9]. Approximately 183.5 Mb sequences were obtained after reassembly by the long-read assembler. After filtering out sequences shorter than 100 bp, approximately 92 Mb sequences with an N50 size of 17.36 Kb were retained (80.70% of the expected genome sequence) and the longest sequence was 326.94 Kb. Approximately 88 Mb sequence scaffolds (76.52% of the expected genome sequence) were allocated to physical contigs and connected by paired-end sequences. Approximately 98 Mb sequences of physical contigs with an N50 size of 312.52 Kb were obtained by connecting sequence scaffolds assigned to the same physical contigs and the longest physical contig was 903.32 Kb. Finally, 38 Mb sequences for chromosome 1, 33 Mb for chromosome 2 and 26 Mb for chromosome 4 were obtained by connecting all physical contig sequences with 50 kb gaps.

To validate the assembled genome sequences, a comparison between assembled chromosomes of rice 93–11 and chromosomes of rice Nipponbare were performed (S1 Results Section D). Most sequence scaffolds were allocated to the corresponding loci on chromosomes by BESs with some gaps. There were 18 loci were assigned to the wrong places because of the repeats on BESs.

## Discussion

We present FGM, a new method for simultaneous physical mapping, whole genome sequencing and *de novo* assembly. This new method accomplishes physical map construction, genome sequencing and sequence integration to the physical map by resolving the same data sets.

### Advantages of this method

During constructing a BAC physical map with fingerprinting, the restriction bands are used as landmarks to determine clone overlap [26]. According to the overlapping algorithm [35, 40], a larger number of total landmarks enables more sensitive determination of clone overlap. For the fingerprinting method with the ABI PRISM® SNaPshot™ Multiplex Kit [26], a maximum of approximately 12,000 bands from 50–500 bp could be distinguished using GS500LIZ (GeneScan™ 500 LIZ® Size Standard, Applied Biosystems) as the size standard, and a maximum of approximately 30,660 bands from 50–1,200 bp could be distinguished using GS1200LIZ (GeneScan™ 1200 LIZ® Size Standard, Applied Biosystems) as the size standard. Compared to the fingerprinting method, optical mapping [31] and nanochannel genome mapping [32] use groups of ordered restriction bands as landmarks and WGP [30] uses sequences as landmarks, and so can significantly increase the resolution. However, all optical mapping, nanochannel genome mapping and WGP, as well as the fingerprinting method, are dependent on restriction enzyme digestion. Usually, they can generate landmarks only from the same group of restriction enzyme digestion each time, integrate only data sets from the same restriction enzyme digestion and generate only physical maps.

Optical mapping and nanochannel genome mapping can be used to generate whole genome ordered restriction/specific sequence motif maps directly using genomic DNA [41, 42] and, in this case, have multiple advantages, such as omitting cloning of large insert DNA fragments, providing potentially very long scaffolds and discerning genome wide DNA methylation profiles [43–45]. However, these maps are not determinative and cannot provide DNA templates for sequence completion. They usually can only be used to validate the well-preassembled sequence and flag the differences. On these physical maps, the landmarks depending on specific restriction enzymes may not be enough to comprehensively align and validate the preassembled sequences of complex genomes. Therefore, other powerful resources are sorely desired for completion of complex genome sequences.

Our method does not require restriction enzyme digestion and can use any sequence as the prefix sequence(s). Many more sequence landmarks (FSs) can be detected using our method. For example, if the FS length is 30 and the prefix sequences are “GGATCC”, “GAATTC”, “TCTAGA” and “CTCGAG”, 464,353 FSs can be detected in the rice genome (*Oryza sativa ssp*. *Japonica* Nipponbare, build 5) and 1,640,376 FSs can be detected in the maize genome (*Zea mays ssp*. *Mays*, version 2, http://www.maizesequence.org). The omission of the restriction digestion step significantly simplifies the experiment, avoiding problems associated with the distribution of restriction sites throughout the genome and enzyme digestion, and reducing cost. FSs are absolute values without errors, different from the restriction digestion-dependent landmarks that could contain errors introduced by experimental conditions, reaction systems, size determination and manipulations. When contiging clones according to FS-sets, it is very easy to determine whether one FS is shared by other clones. Meanwhile, the tolerance value in assembly programs (such as FPC) related to the size resolution of gel or capillary electrophoresis should be set to 0 and so different FSs/bands can be easily distinguished. Most importantly, our method can easily integrate different data sets by selecting the same prefix sequence(s) and generate simultaneously *de novo* physical maps and draft whole genome sequences of complex genomes. The physical map-integrated draft genome sequences provide permanent frameworks and can be completed by targeted sequencing, gap filling and combining the sequences of the same genome produced by any other techniques, such as Illumina/Solexa Genome Analyzer and Pacbio RS system. Many draft genome sequences generated mainly by WGS/NGS were published. Although much cheaper than those generated by CBC method, these draft genome sequences still spent large amounts of money. However, they are usually fragmented unfinished products and cannot be significantly improved by increasing sequencing coverage or by physical maps generated by the restriction enzyme-dependent methods. Without further improvement, these draft genome sequences will have a limited use. Our method has a unique advantage of improving or saving the existing draft genome sequences and finally completing them.

### Parameter determination

An important goal of FGM is to obtain a more accurate genome map and genome sequence while sequencing fewer pools. First, the F-set of each clone in pools should be detected and resolved. There is no doubt that increasing the number of true elements (FSs or k-mers) and decreasing the number of false elements in F-sets of clones increases the accuracy of the physical map and genome sequence.

Sequencing depth is a decisive factor for the detection rate of F-sets for pools and clones. Greater sequencing depth increases the detection rate of F-sets but also increases the number of false elements in F-sets. Other main factors including sequencing errors and pooling dimension also affect the detection rate. Larger pooling dimensions help to remove false elements in clones’ intersected F-sets and to preserve more true elements (FSs or k-mers) in the final F-sets, but result in lower detection rates. Before intersecting the F-sets of pools, elements in F-sets should be filtered because many false elements are imported due to sequencing errors. More than 70% of false elements in the F-sets of pools could be removed at the filtering frequency of 1, increasing the correct rate of F-sets of pools up to 97%. If the sequencing depth for each pool increases, then the filtering frequency of 2 or greater is necessary to be used to filter errors. Pool coverage is another critical factor that affects the ability to resolve clones’ final F-sets. Obviously, 9D pooling produces more accurate F-sets of clones than 6D pooling at the same pool coverage, but it requires sequencing many more pools, increasing cost. It is worthy to note that 2D pooling is much simpler, but also requires sequencing many more pools. As the costs for the construction of sequencing libraries and for NGS reduce, 2D pooling can be considered. Therefore, the determination of pool dimension should be based on a balance between cost and accuracy/simplicity.

The integration of assembled NGS sequences was carried out using a new strategy that allocates assembled sequences to bins on physical contigs, which is different from the previous BAC pooling methods that assembled sequence scaffolds with BAC clones in random [46] or in a minimum tiling path [47]. Most sequence contigs were not only assigned to physical contigs but also ordered and oriented by bin locations. However, errors are still unavoidable. At the level of the physical contig, the orientation of physical contigs can only be confirmed when two or more molecular markers are available on each physical contig. At the level of the sequence contig, the location and orientation of sequence contigs can only be confirmed when paired-end matches spanning close bins are available or when sequence contigs are assigned at a junction of two bins.

Beside the parameters listed above, other parameters such as genome size, read length, FS length and physical map coverage should also be considered. The parameters and suggestions resulted from this work are summarized (S5 Table), and can serve as a reference for future work.

In the experiment conducted with our method, although we used only approximately 3X genome coverage clones and one 500 bp-size sequencing library for NGS, we still obtained a physical map of chromosome 1, 2 and 4 of rice 93–11 containing 452 contigs and 3,364 BAC clones, and obtained approximately 92 Mb sequences and allocated approximately 95.65% (88 Mb) of them to physical contigs. The clone usage is approximately 82.12%, which is much better than the result of fingerprinting technology. Actually, it’s almost impossible to construct a feasible physical map with fingerprinting data of 3X genome coverage clones. Nevertheless, the method may still be improved by using sequencing libraries of various sizes (e.g., 200 bp, 500 bp, 1 kb, 2 kb) and further optimizing the pooling strategy and other parameter combinations.

## Conclusions

In this paper, we present a new method, FGM, which can simultaneously generate *de novo* physical maps and physical map-integrated draft genome sequences using the same NGS data sets. The physical map-integrated draft genome sequences provide permanent frameworks for eventually obtaining high-quality reference sequences by targeted sequencing, gap filling and combining other sequences of the same genomes. Data sets produced by any other techniques from different projects for the same genome can be integrated easily because the FSs and k-mers are absolute values. This feature makes our method unique in improving or saving the existing draft genome sequences generated by WGS/NGS. Compared with the traditional CBC strategy, this method reduces cost, increases efficiency, and improves the compatibility of data when creating physical maps. Compared to WGS/NGS, the assembled sequences can be precisely located, oriented, and connected based on the physical maps, which is important for filling gaps subsequently by map-guided targeted sequencing or by the integration of long Sanger or third-generation sequence reads. This method combines the advantages of CBC and WGS/NGS, avoids their shortcomings, and is expected to be used for a broad range of species.

## Methods

### Simulation and Experimental Validation

Firstly, we inspected many parameters in workflow by simulating to find relative best combination of parameters using the known complete sequences of *Arabidopsis thaliana* ecotype Columbia (TAIR10; http://www.arabidopsis.org/). Then, a group of parameters were used to test an assembly of *Oryza sativa ssp*. *indica* 93–11 (the chromosome 1, 2 and 4). In the experimental test, approximately 3 tiles of BAC clones (covering approximately 115 Mb) were selected from a draft physical map of 93–11. All method details were described in S1 Material and Methods.

### Data availability

The main result was shown in S2 Data. And the raw data were upload to the database of European Nucleotide Archive on EMBL-EBI (https://www.ebi.ac.uk/ena/) [Study accession number: PRJEB12942].

## Supporting Information

S1 Algorithms Supplemental algorithms. (PDF)

S1 Data Pool indexes of experimental testing. Each line defined a pool. The first field is the names of pools, and the other fields were clones in the pool.
(CSV)

S2 Data Main result data. (ZIP)

S1 Material and Methods Details of material and methods. (PDF)

S1 Results More results of this study. (PDF)

S1 Table Quality matrix analysis. The quality matrix was from sequencing data.
(XLS)

S2 Table Pool depth analysis. Different pool depths were analyzed with and without sequencing errors at random and solid pooling strategy respectively.
(XLS)

S3 Table Pool elements frequency analysis. All elements in FS-sets and K-sets were analyzed to find the distribution of true elements in each set.
(XLS)

S4 Table Pool coverage analysis. The genomic coverage of pools was analyzed to find the best pool coverage and to investigate the error rate at each calculating step.
(XLS)

S5 Table Summary of parameters and suggestions. All considered parameters were listed to help to make the best pooling strategy of whole genome sequencing.
(PDF)
