# Supplementary material for: Whole Genome Mapping with Feature Sets from High-Throughput Sequencing Data
Source: PLoS One. 2016 Sep 9;11(9):e0161583. doi: 10.1371/journal.pone.0161583 (PMC5017645; doi:10.1371/journal.pone.0161583)
Supplement: S1 Algorithms — (PDF) [file pone.0161583.s001.pdf]

Algorithms

Yonglong Pan, Xiaoming Wang, Lin Liu, Hao Wang and Meizhong Luo*

National Key Laboratory of Crop Genetic Improvement and College of Life Science and Technology, Huazhong Agricultural University, Wuhan 430070, China

* To whom correspondence should be addressed. E-mail: mzluo@mail.hzau.edu.cn, fax +86 27 87284213

A. About k-mer and feature sequence

The definition of k-mer was adopted from Zerbino and Birney (1). Figure 1 shows the definition of feature sequence (FS) that is the upstream sequence of prefix sequence including plus strain and complementary in read.

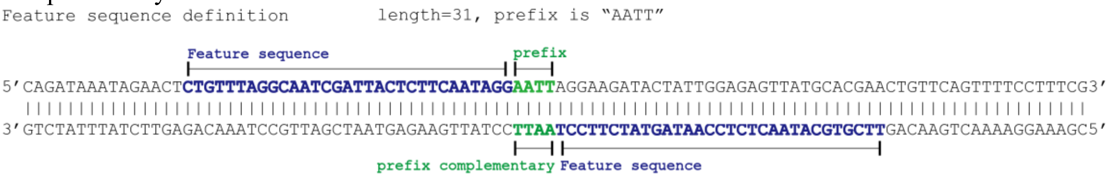

Figure 1. Feature sequence definition. In sequence read, feature sequence (FS) is the upstream sequence of prefix sequence including plus strain and complementary

B. Definition of symbols

There are two sets A and B, then

A ∩ B means that the set of result contains elements in A and in B;

A ∪ B means that the set of result contains elements in A or in B;

A – B means that the set of result contains elements in A not in B;

|A| means the count of elements in A.

There are two code frequency sets A and B, then

A+B means that the code frequency set of result contains elements in A and in B, and the frequency is added for each same element;

A–B means that the code frequency set of result contains elements in A not in B;

If an element is in A and in B, the frequency of this element in A is a, and the frequency in B is b. Then the frequency in result set is $b - a$. If $b - a \leq 0$, then the element will be removed from the result set.

Key(A) means that the set contains all elements in A.

C. Deconvolution and error elimination

For deconvoluting k-mer sets of clones, it's supposed that a clones were constructed to x dimension pools and each dimension contains n pools. The k-mer set of arbitrary pool was presented as $P_{(\kappa,\lambda)}$ ($\kappa<x$, $\lambda<n$) and all k-mer sets of pools combined the set $P = \{P_{(\kappa,\lambda)} | \kappa < x, \lambda < n\}$. There were x pools containing the same clone and their k-mer sets were $\{P_{(\delta)} | \delta<m,\ P_{(\delta)} \in P\}$, then the intersected k-mer set (PKS) of this clone was $C = \bigcap_{\delta=1}^{x} P_{(\delta)}$. For arbitrary clone, its intersected k-mer set was presented as $C_{(\tau)}$ ($\tau<a$). For a given clone in a given pool, the one-lacked union set of the given pool

was obtained by joining all intersected k-mer sets of all clones excluding the given clone. Setting all one-lacked union sets of all pools containing the given clone to $\{P(\nu)|\,\nu<x\}$, final k-mer set (FKS) of the given clone was $C = C_{(\tau)} - \bigcap_{\nu=1}^{x} P_{(\nu)}$.

As for feature sequence set of clone, the set of feature sequences is a subset of k-mer set. So, final feature sequence sets of clones can be obtained by calculating sets with the same algorithm above.

D. Balancing the pool's F-set

We give a new definition - code frequency set (CF-set) which is a set that contains elements and frequency of each element. For example, a code frequency set contains elements $\{(c_1, f_1), (c_2, f_2), (c_3, f_3)\ldots (c_n, f_n)\}$. The code frequency (c_n, f_n) means that the element c_n occurs f_n times in the code frequency set.

Suppose that there are n clones in a pool. First of all, the frequency of each element in clone's k-mer set is set to 1. When uniting the clones' intersected k-mer sets (C), the sum of all frequency of the same element is the new frequency for this element in the union pool's k-mer code frequency set (pU-CF-set). Next, when comparing the union pool's k-mer set to screened k-mer set of the same pool, the frequency of each element in a set is set to 2 to get a new code frequency set if the set contains elements in screened k-mer set but not in union pool's k-mer set. Finally, the new code frequency set is added to the pU-CF-set to get the filled union of pool's k-mer code frequency set (fU-CF-set).

If a give clone's k-mer set is C, then a code frequency set of this clone is C' which contains all elements in C and the frequency of each element is 1. For a given pool containing this clone, if this pool's fU-CF-set is P, then the code frequency set excluding the given clone is $C' = P - C$. Key(C') is the set of the one-lacked union set of the given pool.

E. Combination strategy

For large libraries, it's too complicated to put all clones into a cube. Generally, clones are stored in 384-well or 96-well plates. We could construct sub-pools for each plate. For example (Figure 2), in 384-well plate, each row is split to 3 groups, and each column is split to 2 groups. Each group contains 8 contiguous clones as a sub-pool. Then all sub-pools are put into a cube to construct pools to sequence. When deconvoluting, the F-sets of sub-pools are screened and then clones' F-sets are obtained from sub-pools F-sets. This strategy would increase efficiency and reduce complex of pools' construction.

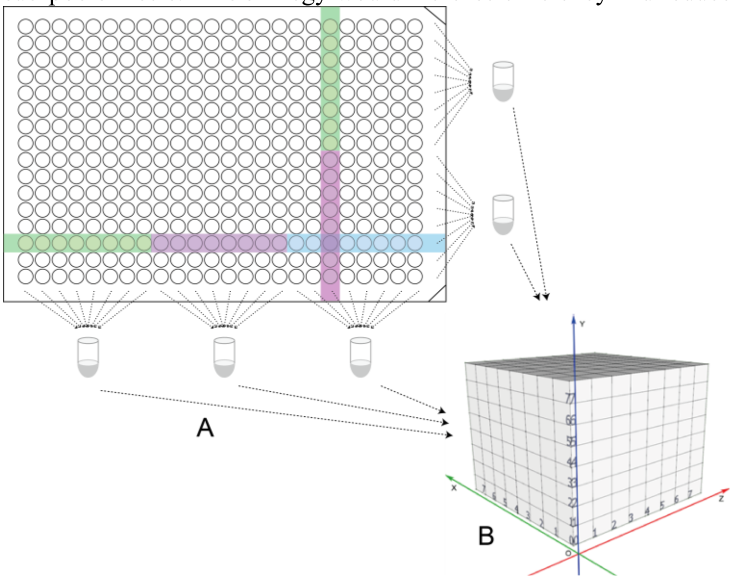

Figure 2. Combination strategy for large library.

Reference

1. Zerbino DR, Birney E. 2008. Velvet: algorithms for de novo short read assembly using de Bruijn graphs. *Genome Res* **18**(5): 821-829.
