# Supplementary material for: Whole Genome Mapping with Feature Sets from High-Throughput Sequencing Data
Source: PLoS One. 2016 Sep 9;11(9):e0161583. doi: 10.1371/journal.pone.0161583 (PMC5017645; doi:10.1371/journal.pone.0161583)
Supplement: S1 Material and Methods — (PDF) [file pone.0161583.s004.pdf]

Materials and Methods

Yonglong Pan, Xiaoming Wang, Lin Liu, Hao Wang and Meizhong Luo*

National Key Laboratory of Crop Genetic Improvement and College of Life Science and Technology, Huazhong Agricultural University, Wuhan 430070, China

* To whom correspondence should be addressed. E-mail: mzluo@mail.hzau.edu.cn, fax +86 27 87284213

A. Simulation *in silico*

We inspected many parameters in workflow by simulation using the known complete sequences of *Arabidopsis thaliana* ecotype Columbia (TAIR10; http://www.arabidopsis.org/) to find the relative best combination of parameters.

1. Data preparation

Before simulation, a complete genome sequence was selected as a reference that was used as the basis for all simulations. The complete *Arabidopsis thaliana* ecotype Columbia sequence was used to simulate BAC library construction, clone pool creation, and pool sequencing by NGS *in silico*. The gaps in the genome sequences denoted as "N" were randomly filled in by the four nucleotides ("A", "T", "G" or "C").

When constructing the BAC library, the genome sequences were partially digested by *Bam*HI *in sillico*. Firstly, a random integer was produced to select the chromosome. Then, all recognized sites of *Bam*HI on this chromosome were found and two random sites were selected to produce the sequence fragments. If the sequence fragment was between 60 kb and 300 kb, it was selected and inserted into the BAC vector (1) as a BAC clone. The sizes of insert fragments followed a normal distribution, with an average of 137.42 kb and a variance of 417.54 kb^2.

2. Pools Construction

We placed a cube of BAC clones in a right-hand rectangular coordinate system (Figure 1). Each clone (corresponding to each cell) could be located by its coordinates on the three axes (x, y, and z). When a clone cube is defined, either solid pooling or random pooling can be used in the next step.

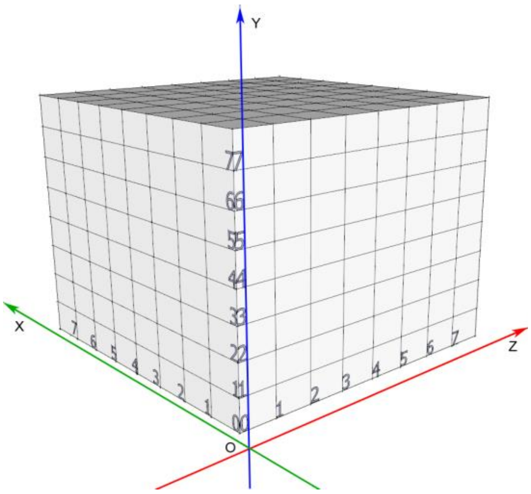

Figure 1. Pooling cube in a rectangular coordinate system. A clone in the cube can be located by its three coordinate values (x, y, z) in the system.

For solid pooling, 9 types of pool arrays (Figure 2) were classified: perpendicular crossing pools including PX, PY and PZ, oblique crossing pools including OX, OY and OZ and angle crossing pools including AX, AY and AZ. The pools of PX, PY, PZ, OX, OY and OZ correspond to side pool (2), plate pool (3), face pool (FP), row pool (RP), diagonal pool (2) and column pool (CP), respectively, as addressed in earlier studies (4-6). Clones with the same color and texture (Figure 2) in each type belong to the same pool. All the pools contain the same number of BAC clones, so that each pool contains a close sum of genome segmental size. The pool dimension represents the number of types of pool arrays selected when designing pools. For example, 3 dimensional (3D) pools could include PX, PY and PZ or 3 arbitrary types out of the 9 types.

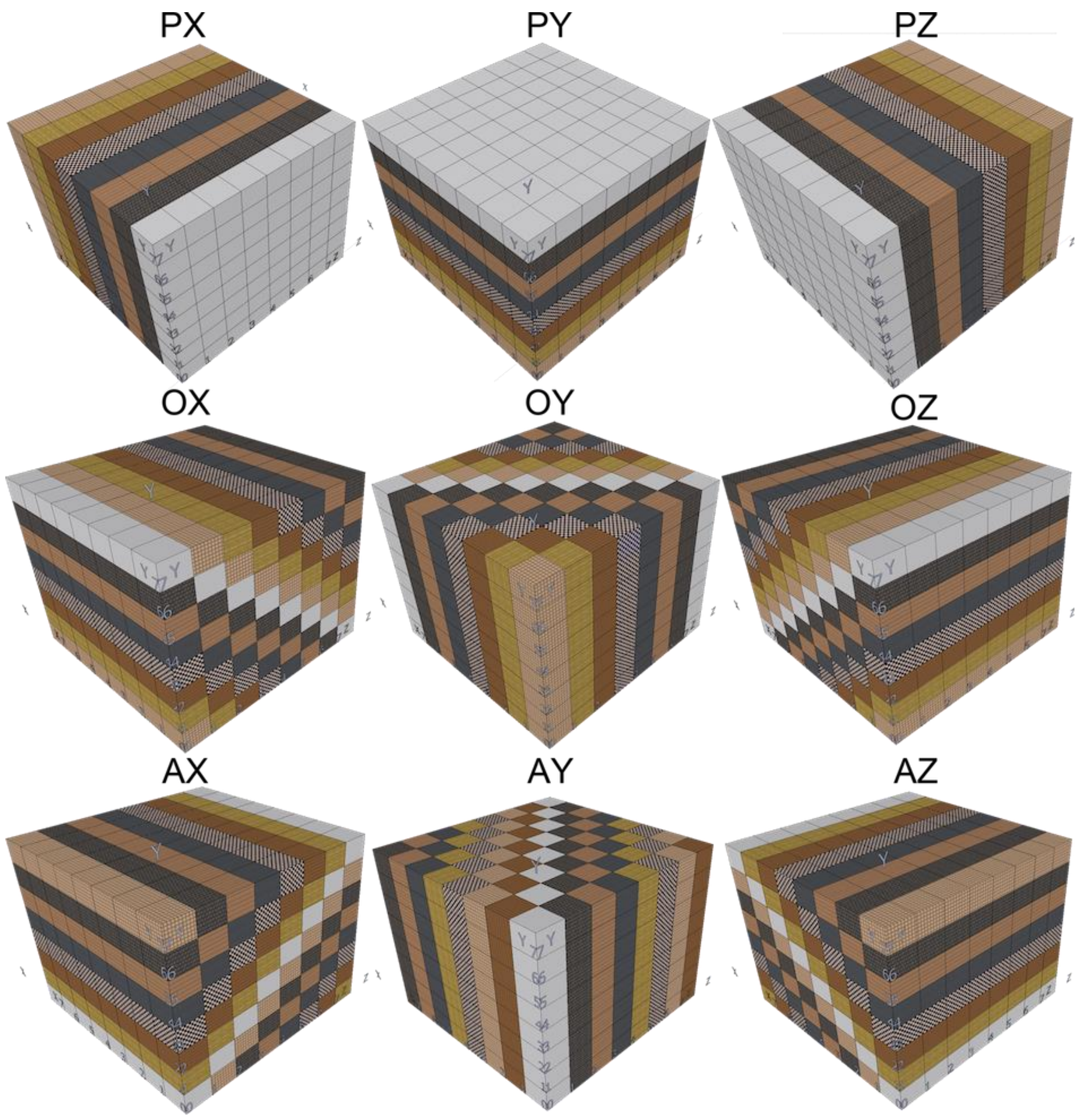

Figure 2. Solid pooling strategy definition. The clones with the same color and texture belong to the same pool in each type.

For random pooling, 3D pools are created by perpendicularly crossing pools defined in the solid pooling strategy, 6 dimensional (6D) pools are created by combining the existing 3D pools with other 3D pools of randomly reshuffled clones, while 9 dimensional (9D) pools are created by combining the existing 6D pools with 3D pools of randomly reshuffled clones.

For simulations, 3D, 6D and 9D pooling dimensions and the solid and random pooling strategies were investigated to determine the best pool dimension and pooling strategy, respectively. The number of BAC clones and the genome coverage of BAC libraries were variables in different simulations.

3. Sequencing of the pools' DNA

All pools' DNA was paired-end sequenced in the simulation. Firstly, a random integer was produced to select a chromosome. Then, a new random integer between 1 and the length of the chromosome was produced to select the sequencing start site. Next, the third random integer between 450 and 500 was produced to select the length of a sequence in the sequencing library. Finally, two sequences of 100 bp at the ends of the library fragment were the paired-end reads. All produced reads

was 100 bp in length; the sequencing library size for paired-end sequencing ranged between 450 and 500 bp. To obtain sequences close to experimental sequences from NGS, a quality matrix was introduced into the sequencing simulation.

The quality matrix was generated using more than 100 billion sequence reads from Illumina/Solexa Genome Analyzer (Supplemental Table S1). Each read was 100 bp in length, and the base site was indexed from 1 to 100. The quality frequency on each base site of all reads was counted and recorded in the quality matrix. All quality values of every site were indexed in ascending order. Suppose that i ($1 \le i \le 100$) is an arbitrary index of a given site; Q_i (the value is queried in the quality matrix) is the sequencing quality of index i; $\sum Q$ is the total frequency of the given site; $\sum_1 Q_i$ is the sum frequency from index 1 to index i. When sequencing a given site of a read by NGS in a simulation, a random value R between 0-1 is generated; if the value of $R \times \sum Q$ is between $\sum_1 Q_{i-1}$ and $\sum_1 Q_i$, then the quality of the given site is Q_i. The error probability of this site is calculated according to the obtained Q_i (7), and then a new random value R' is generated; if R' is larger than the calculated error probability, then this base at the given site is the original base; otherwise, the base is randomly replaced by other bases. For the NGS *in silico* without errors, all given sites are the original bases.

4. Deconvolution and error elimination

All reads of each pool were screened to find all FSs, each of which was the 31 bp upstream of the prefix sequences including “GGATCC” and “GAATTC”. The FS-set of a pool represented the combination of all FSs of the pool. The FSs occurring fewer times (once in this paper) were removed from the FS-sets. Next, the intersected FS-set of each clone was obtained by intersecting the FS-sets of all pools containing this clone. For a given clone in a given pool, all FS-sets of clones excluding the given clone in the given pool were joined to a union set. To eliminate false FSs, the intersection of all union sets obtained above was subtracted from the FS-set of the given clone to obtain the final FS-set of the given clone. The final FS-sets of all clones were used to construct the physical map.

All reads of each pool were screened again to find all K-sets of pools. All K-sets of clones were obtained, and used to locate and connect sequence contigs.

5. Building of physical map

All FSs were indexed by assigning a positive integer to the same FS. According to the indexes, the FS-sets of clones were converted to “.size” files compatible with the FingerPrinted Contig (FPC) program (8). Contiging was performed using FPC (v9.4) with a tolerance value of 0 (this value must be set to 0). Different cut-off values were tested to find the best arrangement of clone overlap. Next, a DQ analysis was performed to remove Q-clones and split problematic contigs until no contigs contained more than 5 Q-clones. Finally, a list of contigs with the corresponding order of clones was obtained by FPC.

6. Integration of sequences and physical map

According to the physical map, each physical contig was split to many bins that contained k-mers from overlapping clones (Figure 3). The sequence reads of the pools were assembled by a short-read assembler such as velvet (9), SOAPdenovo (10). Next, all contigs from the output of the short-read assembler were re-assembled by a long-read assembler such as Phrap (http://www.phrap.org), Phusion (11), or PCAP (12). In this study, the software of velvet and Phrap were employed. Each contig from the output of the long-read assembler was split into a k-mer set and assigned to bins based on the best

intersection between the k-mer sets of the contig and each bin.

Paired-end alignment between reads of pools from NGS and the long sequence contigs was performed with Bowtie 2 (13). The match identity was greater than 97% because the error rate was 2.46%. Sequence contigs that were paired-end matched and allocated in the same or nearby bins were connected to longer sequence scaffolds. The 550 bp of "N" were inserted between two sequence contigs that were allocated to the same bin but not oriented. All sequence scaffolds on the same physical contig were connected to the sequence of this physical contig (Figure 3). Additionally, 50 kb of "N" were inserted into each gap between two neighboring sequences of physical contigs.

Finally, the sequences of physical contigs were allocated to chromosomes and oriented by known markers (http://www.arabidopsis.org/) on clones.

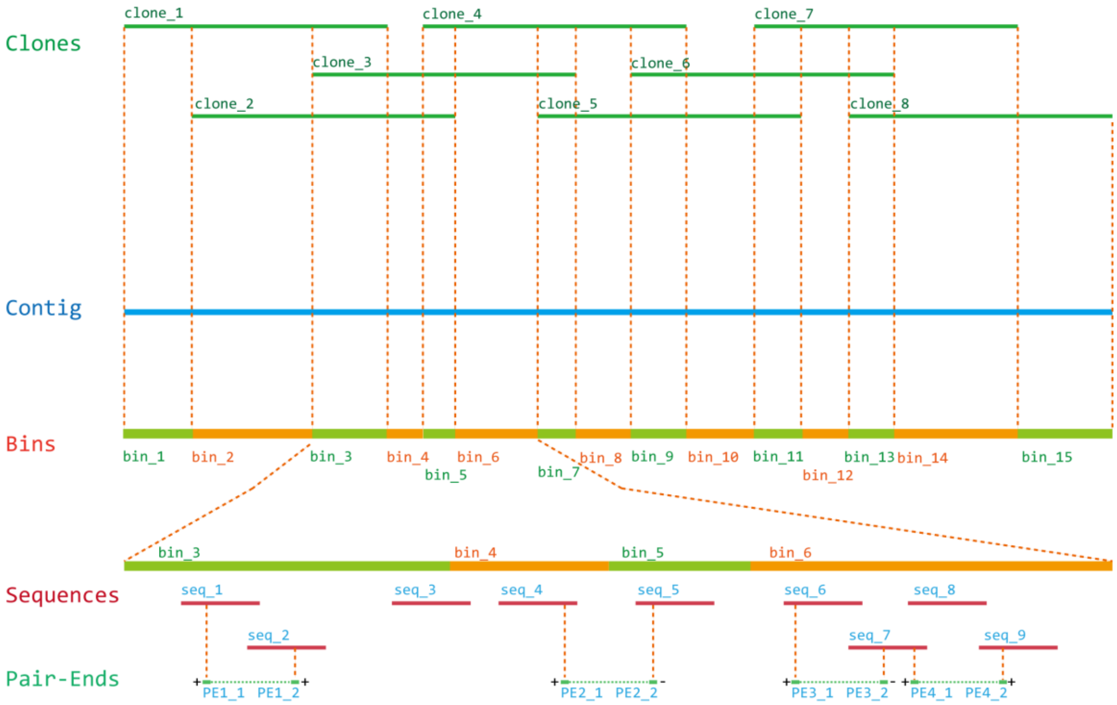

Figure 3 Splitting a contig into bins according to the clones' order in the physical contig. The k-mer set of each bin was derived from the intersections or differences among overlapping clones. Assembled sequences were allocated to the best bins. Paired-end sequences connected assembled sequences located at the same or nearby bins to larger sequences. The orientation of connected sequences was determined by the sequence loci and the directions of paired-end alignments. The red blocks labeled with the prefix "seq" indicate assembled sequences from the long-read assembler. The prefix "PE" labels the paired-ends. The symbol "+" or "-" indicates the directions of paired-end alignments.

7. Validation

In simulations, the original and assembled chromosomes were compared using the SyMAP V4.0 program (14, 15) to validate the result.

B. Experimental testing

1. Data preparation

The complete sequences of chromosome 1, 2 and 4 of *Oryza sativa ssp. japonica* Nipponbare (Build 5; http://rgp.dna.affrc.go.jp) was used a reference for the testing of chromosome 1, 2 and 4 of *Oryza sativa ssp. indica* 93-11. Approximately 3 tires of BAC clones (covering approximately 115 Mb) were selected from a draft physical map of 93-11 (16).

2. Pools Construction

For this test, we selected 4,096 clones, 4,064 of which were mapped on chromosome 1, 2, and 4 of a draft 93-11 physical map. These 4,096 clones were then put into a 16X16X16 cube and assigned to 96 pools of 6 types (PX, PY, PZ, OY, AX and AZ) using the solid pooling strategy. Each pool contained 16X16=256 clones.

3. DNA extraction of pools

For experimental validation, 4,096 clones were picked into 96-well culture plates containing 1,000 μl freezing media in each well (17), cultured for 18 hours at 37°C, and then stored at -80°C. In each 96-well culture plate, 8 successive clones in columns or rows were combined into a primary pool. If there were not 8 clones in a column or row of the plate, then clones from the next plate were mixed into the primary pool. Each primary pool contained 8X 150 μl of each well (total 1,200 μl) and was stored in another 96-well culture plate. According to the pool design, every 32 primary pools were replicated to a new 96-well culture plate, and each well contained 100 μl of liquid from the primary pool and 1,100 μl 2×YT media. The primary pools stored in the wells from columns 1 to 4 formed a pool block, and those stored in the wells from columns 9 to 12 formed another pool block. Therefore, each pool plate stored two pool blocks, each of which contained 256 clones. Pool plates were cultured for 18 hours at 37°C. Finally, each pool block was replicated for 15 copies by mixing 40 μl of the liquid of each pool block well with 1,000 μl 2×YT media; the 15 copies were then cultured for 18 hours at 37°C and used to extract plasmid DNA using the QIAGEN® Large-Construct Kit.

4. Sequencing of the pools' DNA

Plasmid DNAs of all 96 pools were paired-end sequenced by Illumina Genome Analyzer. The DNA fragments in the sequencing library were approximately 500 bp in length. Each read was 100 bp; the accumulated read length of each pool was more than 10^9 bp, representing a pool coverage of more than 30X.

5. Deconvolution and error elimination

All reads of each pool were screened to find all FSs, each of which was the 31 bp upstream of the prefix sequences including "GGATCC" and "GAATTC". All reads of each pool were screened to find all FS-sets and K-sets of pools and all FS-sets and K-sets of clones were obtained using the same algorithm in simulations, and used to locate and connect sequence contigs.

6. Building of physical map

All FSs of clones were converted to ".size" files compatible with the FPC program using the same algorithm in simulations. Contiging was performed using FPC with a tolerance value of 0. Different cut-off values were tested to find the best arrangement of clone overlap. A DQ analysis was performed to remove Q-clones and split problematic contigs until no contigs contained more than 5 Q-clones. Finally, a list of contigs with the corresponding order of clones was obtained by FPC.

7. Integration of sequences and physical map

In the experiment, because no known markers were located on clones and because the genome sequence of Nipponbare is more complete than that of 93-11, we used 93-11 BESs instead of markers to map the physical contigs to the chromosomes of Nipponbare.

8. Validation

The reference of chromosome 1, 2 and 4 of *Oryza sativa ssp. japonica* Nipponbare and the assembled chromosomes were compared using the SyMAP V4.0 program (14, 15) to validate the result. A re-assembly of physical map was performed using the original fingerprint data for the same 93-11 clones used in the testing experiment.

Reference

1. Shi X, Zeng H, Xue Y, & Luo M (2011) A pair of new BAC and BIBAC vectors that facilitate BAC/BIBAC library construction and intact large genomic DNA insert exchange. *Plant methods* 7:33.
2. Encode Project Consortium*, et al.* (2012) An integrated encyclopedia of DNA elements in the human genome. *Nature* 489(7414):57-74.
3. Applied Biosystems (2011) *Applied Biosystems 3730 and 3730xl DNA Analyzers* (Applied Biosystems).
4. Klein PE*, et al.* (2000) A High-throughput AFLP-based Method for Constructing Integrated Genetic and Physical Maps: Progress Toward a Sorghum Genome Map. *Genome Research* 10:19.
5. Yim Y-S*, et al.* (2007) A BAC pooling strategy combined with PCR-based screenings in a large, highly repetitive genome enables integration of the maize genetic and physical maps. *BMC Genomics* 8.
6. Wu X*, et al.* (2008) Genetic marker anchoring by six-dimensional pools for development of a soybean physical map. *BMC Genomics* 9:13.
7. Cock PJ, Fields CJ, Goto N, Heuer ML, & Rice PM (2010) The Sanger FASTQ file format for sequences with quality scores, and the Solexa/Illumina FASTQ variants. *Nucleic Acids Res* 38(6):1767-1771.
8. Soderlund C, Longden I, & Mott R (1997) FPC: a system for building contigs from restriction fingerprinted clones. *Computer applications in the biosciences : CABIOS* 13(5):523-535.
9. Zerbino DR & Birney E (2008) Velvet: algorithms for de novo short read assembly using de Bruijn graphs. *Genome Res* 18(5):821-829.
10. Li R, Li Y, Kristiansen K, & Wang J (2008) SOAP: short oligonucleotide alignment program. *Bioinformatics* 24(5):713-714.
11. Mullikin JC & Ning Z (2003) The phusion assembler. *Genome Res* 13(1):81-90.
12. Huang X, Wang J, Aluru S, Yang SP, & Hillier L (2003) PCAP: a whole-genome assembly program. *Genome Res* 13(9):2164-2170.
13. Langmead B & Salzberg SL (2012) Fast gapped-read alignment with Bowtie 2. *Nature methods* 9(4):357-359.
14. Soderlund C, Nelson W, Shoemaker A, & Paterson A (2006) SyMAP: A system for

discovering and viewing syntenic regions of FPC maps. *Genome Res* 16(9):1159-1168.

15. Soderlund C, Bomhoff M, & Nelson WM (2011) SyMAP v3.4: a turnkey synteny system with application to plant genomes. *Nucleic Acids Res* 39(10):e68.

16. Pan Y*, et al.* (2014) Comparative BAC-based physical mapping of Oryza sativa ssp. indica var. 93-11 and evaluation of the two rice reference sequence assemblies. *The Plant journal* 77(5):795-805.

17. Luo M & Wing RA (2003) An improved method for plant BAC library construction. *Methods in molecular biology* 236:3-20.
