# Supplementary material for: Whole Genome Mapping with Feature Sets from High-Throughput Sequencing Data
Source: PLoS One. 2016 Sep 9;11(9):e0161583. doi: 10.1371/journal.pone.0161583 (PMC5017645; doi:10.1371/journal.pone.0161583)
Supplement: S1 Results — (PDF) [file pone.0161583.s005.pdf]

Results

Yonglong Pan, Xiaoming Wang, Lin Liu, Hao Wang and Meizhong Luo*

National Key Laboratory of Crop Genetic Improvement and College of Life Science and Technology, Huazhong Agricultural University, Wuhan 430070, China

* To whom correspondence should be addressed. E-mail: mzluo@mail.hzau.edu.cn, fax +86 27 87284213

A. Validation of the in silico BAC library

An *in silico* BAC library was generated. For this, genome sequences were partially digested by *Bam*HI; sequence fragments between 60kb and 300kb were selected and inserted into vector (1). The lengths of insert fragments followed normal distribution with average 137.42kb and variance 417.54, based on a maize BAC library constructed by our laboratory (2). Ten thousand BAC clones were generated randomly in *silico* and the frequency distribution of insert size was analyzed (Figure 1). The insert sizes of clones agreed with the designed distribution.

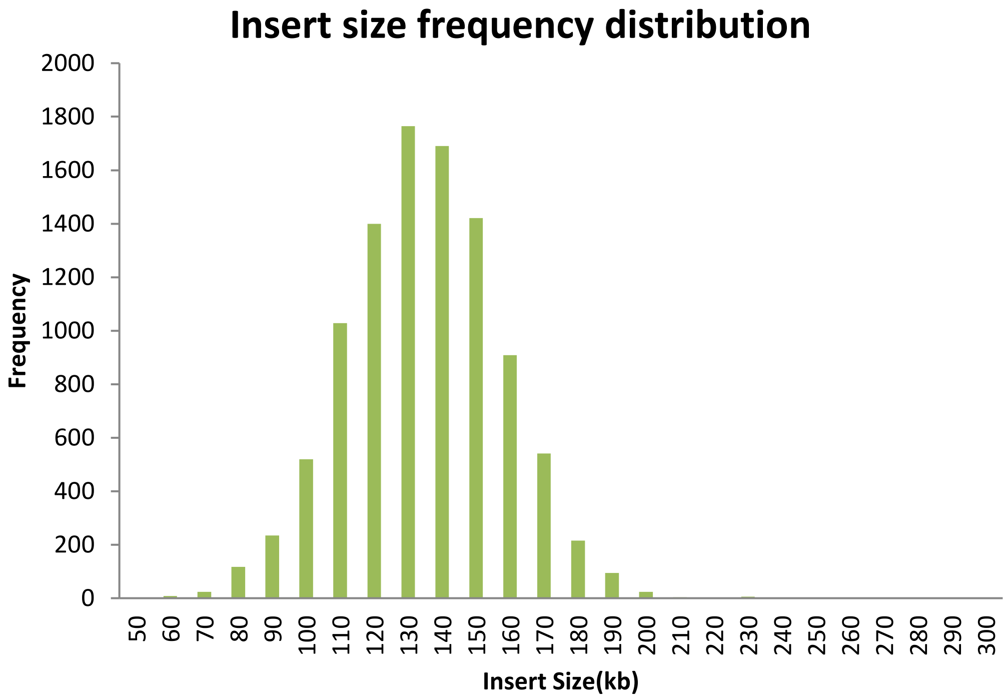

Figure 1. Insert size frequency distribution *in silico*

B. Sequence quality

A matrix of sequence quality was generated according to more than 100 billion sequence reads from Illumina/Solexa Genome Analyzer (Supplemental Table S1). Each read was 100 bp in length, and the base site was indexed from 1 to 100. The quality frequency on each base site of all reads was counted and recorded in the quality matrix. All quality values of every site were indexed in ascending order. Suppose that i is an arbitrary index of a given site; Q_i is the sequencing quality of index i; $\sum Q$ is the total frequency of the given site; $\sum_1 Q_i$ is the sum frequency from index 1 to index i. When sequencing a given site of a read by NGS in a simulation, a random value R between 0-1 is generated; if the value of $R \times \sum Q$ is between $\sum_1 Q_{i-1}$ and $\sum_1 Q_i$, then the quality of the given site is Q_i. The error probability of this site is calculated according to the obtained Q_i (3), and then a new random value R' is generated; if R' is larger than the calculated error probability, then this base at the given site is the original base; otherwise, the base is randomly replaced by other bases.

We counted the number of qualities at each read site, and then normalized the numbers. Figure 2 shows the normalized quality matrix. In read site from 1st to 100th, the bar height reflects the related count at the same read site with different qualities.

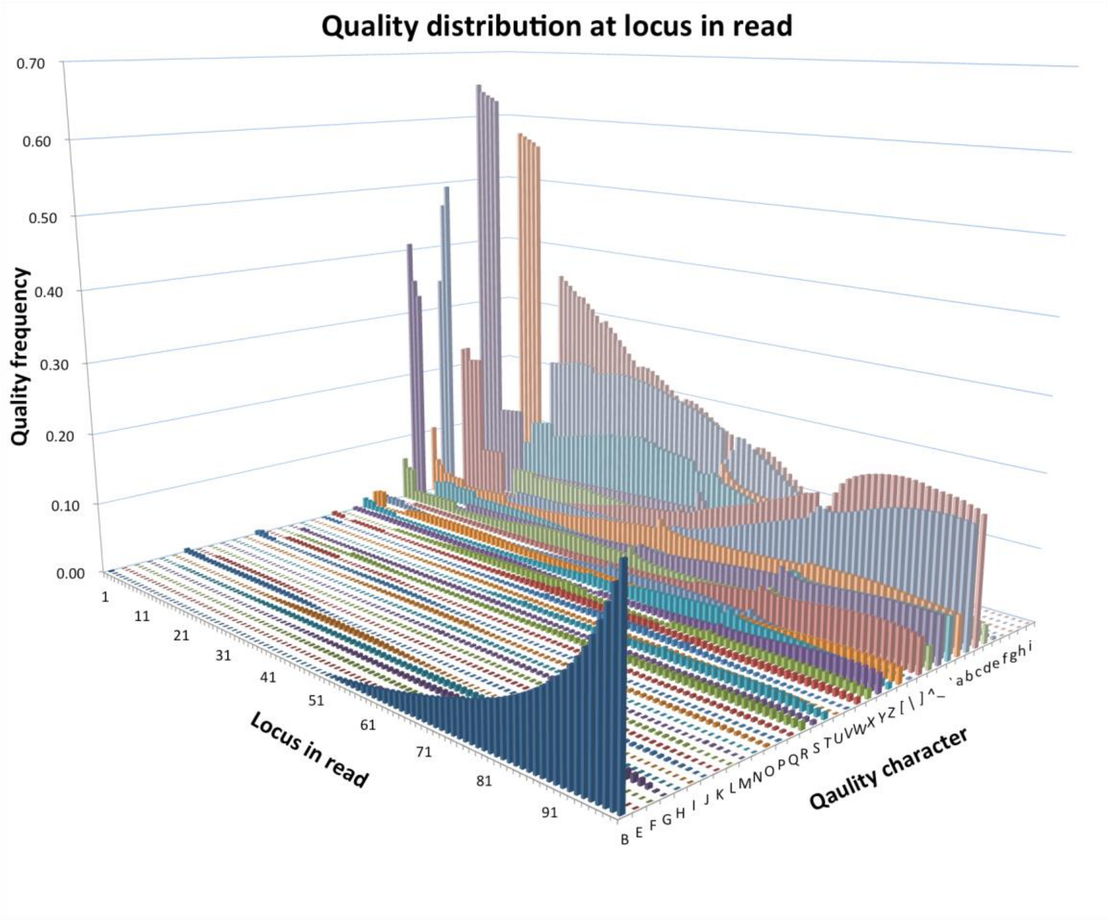

Figure 2. Quality frequency distribution of experiment at each base site in normalization. The bar height is the related count of the same read site with different qualities. The character of quality was the quality in fastq format. Locus in read is the location in NGS sequence read from 1st to 100th.

From the quality matrix, the average error rate at each locus in read was calculated and showed in Figure 3. It is shown that the error rate was increasing with ascending read site number. The average error rate of all reads was 2.46%, which was shown as the read horizon line in Figure 3.

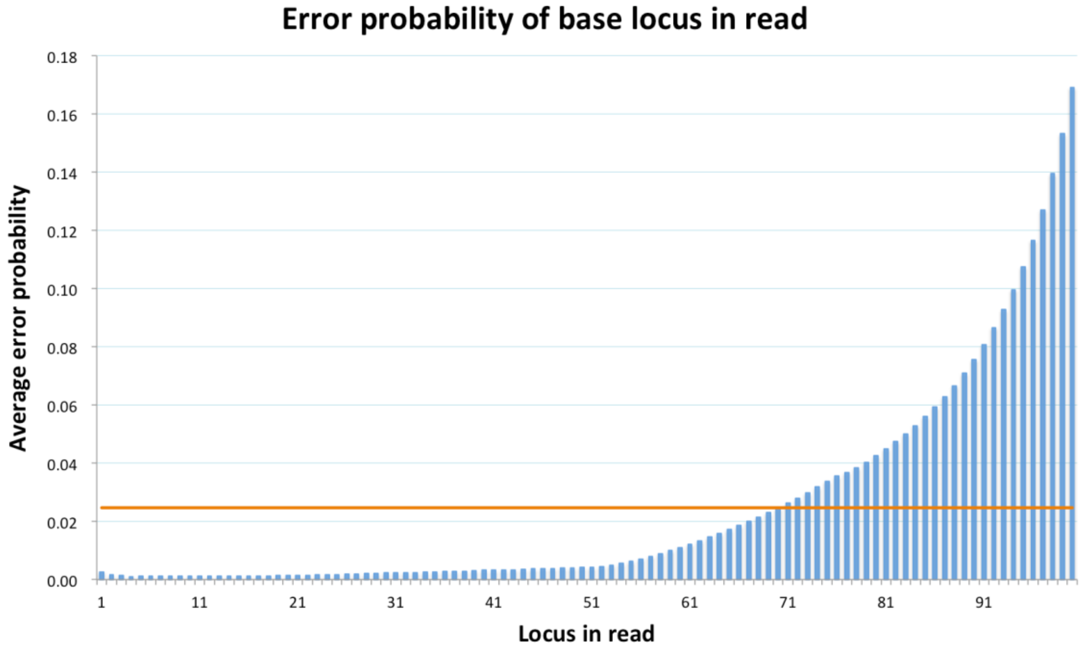

Figure 3. Average error rate of experiment result at each base site in reads. The orange line is the average error probability of all bases.

To validate the simulated sequencing results, about 456 million reads were generated with the quality matrix above; the normalized quality distribution agreed with the expected quality matrix above (Figure 4 and Figure 5).

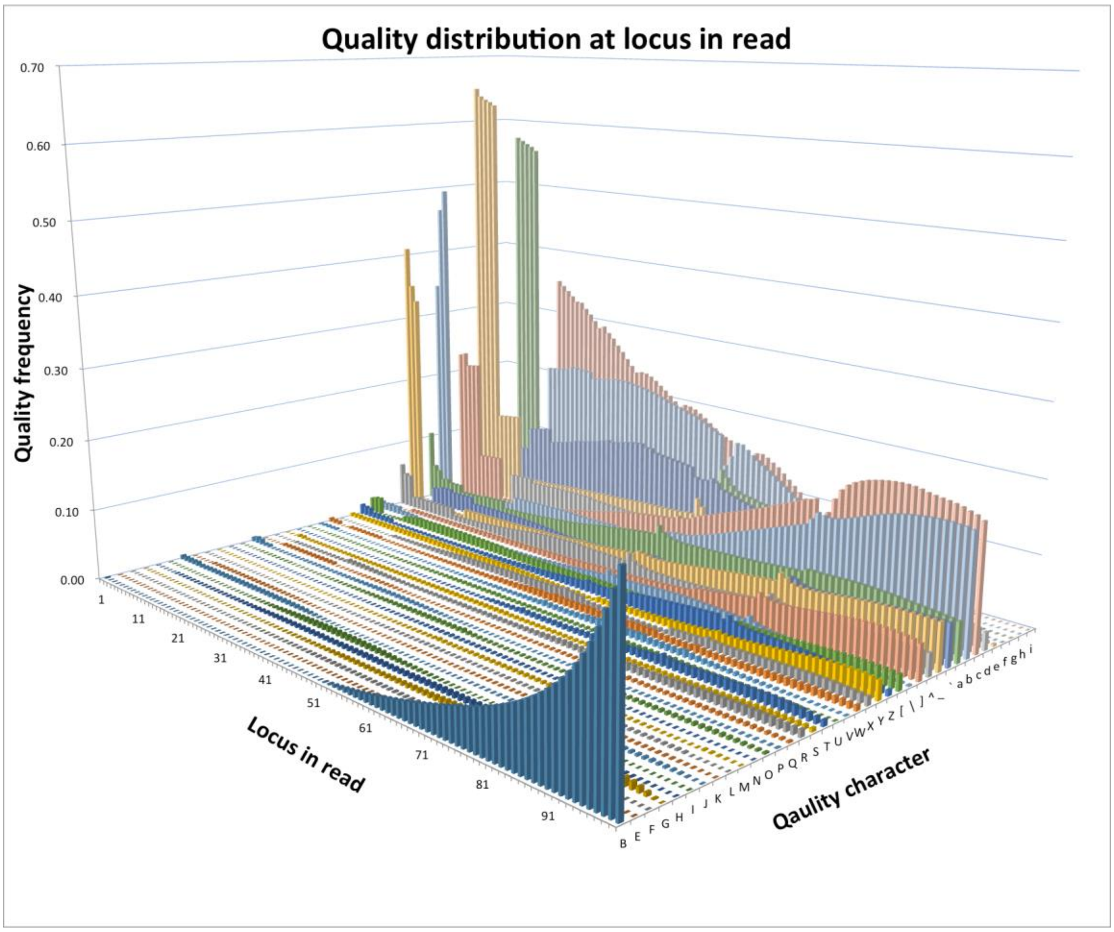

Figure 4. Quality frequency distribution of simulation at each base site in normalization. The bar height is the related count at the same read site with different qualities. The character of quality was the quality in fastq format. Locus in read is the location in NGS sequence read from 1st to 100th.

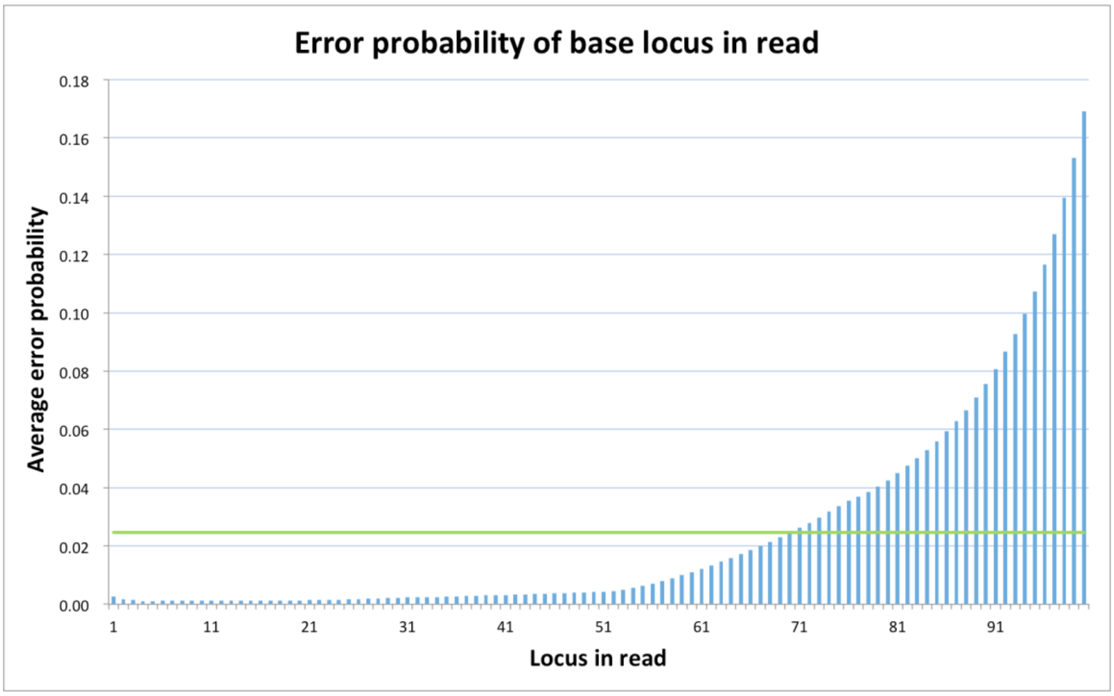

Figure 5. Average error rate of simulation result at each base site in reads. The green line is the average

error probability of all bases.

C. Simulation result

Simulated integration was completed when pool coverage was set to 0.30 for 6D pool. About 270Mb sequences were obtained after reassembled by long-read assembler, but many sequences were very short. Sequences shorter then 100bp were filtered and the remaining sequences were allocated to bins. Then 103.1Mb sequences were allocated and 109.9Mb sequences were obtained after connecting sequence contigs with paired-end sequences. When connecting contigs located on chromosomes, 50Kb “N”s were filled into the gaps. At last, 118.4Mb sequences of 5 chromosomes were obtained.

To validate the chromosome sequences, a comparison between original chromosomes and assembled chromosomes (Figure 4 in Manuscript and Figure 6 in this section) was performed with the program Symap V4.0 (4,5). As the circle view of all chromosomes shown (Figure 4A in Manuscript), most sequences were mapped to the right locations. Only one sequence segment of chromosome 4 was located in error to chromosome 1. The sequences of chloroplast and mitochondria were lost because no markers existed on clones belong to them. A full view of alignments between all chromosomes was shown in dot-plot view (Figure 4B in Manuscript).

Looking at the detail comparison results of all chromosomes (Figure 6), some sequence segments were found to be inversely allocated on physical contigs. This is because only one genetic marker was available. On chromosome 1, there was an expanded region from 27.6Mb to 28.87Mb in the simulated sequence; this region was an error allocation of the sequence from chromosome 4. Some contigs were not assembled in the simulated sequence because they did not contain any genetic markers. In this case, the simulated sequence showed contractions at the corresponding regions.

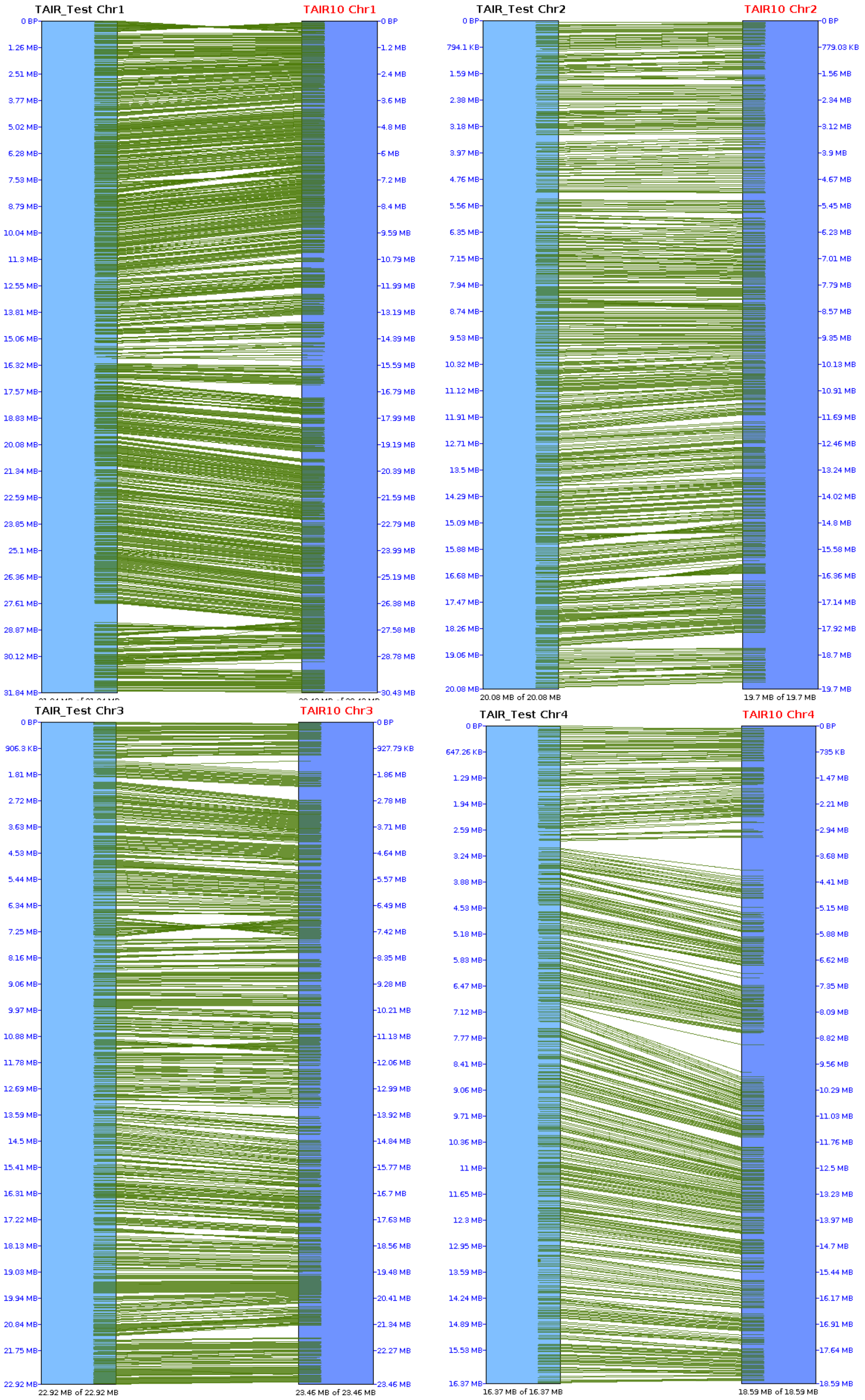
TAIR_Test Chr1
TAIR10 Chr1
0 BP
1.26 MB
2.51 MB
3.77 MB
5.02 MB
6.28 MB
7.53 MB
8.79 MB
10.04 MB
11.3 MB
12.55 MB
13.81 MB
15.06 MB
16.32 MB
17.57 MB
18.83 MB
20.08 MB
21.34 MB
22.59 MB
23.85 MB
25.1 MB
26.36 MB
27.61 MB
28.87 MB
30.12 MB
31.84 MB
0 BP
1.2 MB
2.4 MB
3.6 MB
4.8 MB
6 MB
7.2 MB
8.4 MB
9.59 MB
10.79 MB
11.99 MB
13.19 MB
14.39 MB
15.59 MB
16.79 MB
17.99 MB
19.19 MB
20.39 MB
21.59 MB
22.79 MB
23.99 MB
25.19 MB
26.38 MB
27.58 MB
28.78 MB
30.43 MB
TAIR_Test Chr2
TAIR10 Chr2
0 BP
794.1 KB
1.59 MB
2.38 MB
3.18 MB
3.97 MB
4.76 MB
5.56 MB
6.35 MB
7.15 MB
7.94 MB
8.74 MB
9.53 MB
10.32 MB
11.12 MB
11.91 MB
12.71 MB
13.5 MB
14.29 MB
15.09 MB
15.88 MB
16.68 MB
17.47 MB
18.26 MB
19.06 MB
20.08 MB
0 BP
779.03 KB
1.56 MB
2.34 MB
3.12 MB
3.9 MB
4.67 MB
5.45 MB
6.23 MB
7.01 MB
7.79 MB
8.57 MB
9.35 MB
10.13 MB
10.91 MB
11.69 MB
12.46 MB
13.24 MB
14.02 MB
14.8 MB
15.58 MB
16.36 MB
17.14 MB
17.92 MB
18.7 MB
19.7 MB
20.08 MB of 20.08 MB
19.7 MB of 19.7 MB
TAIR_Test Chr3
TAIR10 Chr3
0 BP
906.3 KB
1.81 MB
2.72 MB
3.63 MB
4.53 MB
5.44 MB
6.34 MB
7.25 MB
8.16 MB
9.06 MB
9.97 MB
10.88 MB
11.78 MB
12.69 MB
13.59 MB
14.5 MB
15.41 MB
16.31 MB
17.22 MB
18.13 MB
19.03 MB
19.94 MB
20.84 MB
21.75 MB
22.92 MB
0 BP
927.79 KB
1.86 MB
2.78 MB
3.71 MB
4.64 MB
5.57 MB
6.49 MB
7.42 MB
8.35 MB
9.28 MB
10.21 MB
11.13 MB
12.06 MB
12.99 MB
13.92 MB
14.84 MB
15.77 MB
16.7 MB
17.63 MB
18.56 MB
19.48 MB
20.41 MB
21.34 MB
22.27 MB
23.46 MB
22.92 MB of 22.92 MB
23.46 MB of 23.46 MB
TAIR_Test Chr4
TAIR10 Chr4
0 BP
647.26 KB
1.29 MB
1.94 MB
2.59 MB
3.24 MB
3.88 MB
4.53 MB
5.18 MB
5.83 MB
6.47 MB
7.12 MB
7.77 MB
8.41 MB
9.06 MB
9.71 MB
10.36 MB
11 MB
11.65 MB
12.3 MB
12.95 MB
13.59 MB
14.24 MB
14.89 MB
15.53 MB
16.37 MB
0 BP
735 KB
1.47 MB
2.21 MB
2.94 MB
3.68 MB
4.41 MB
5.15 MB
5.88 MB
6.62 MB
7.35 MB
8.09 MB
8.82 MB
9.56 MB
10.29 MB
11.03 MB
11.76 MB
12.5 MB
13.23 MB
13.97 MB
14.7 MB
15.44 MB
16.17 MB
16.91 MB
17.64 MB
18.59 MB
16.37 MB of 16.37 MB
18.59 MB of 18.59 MB

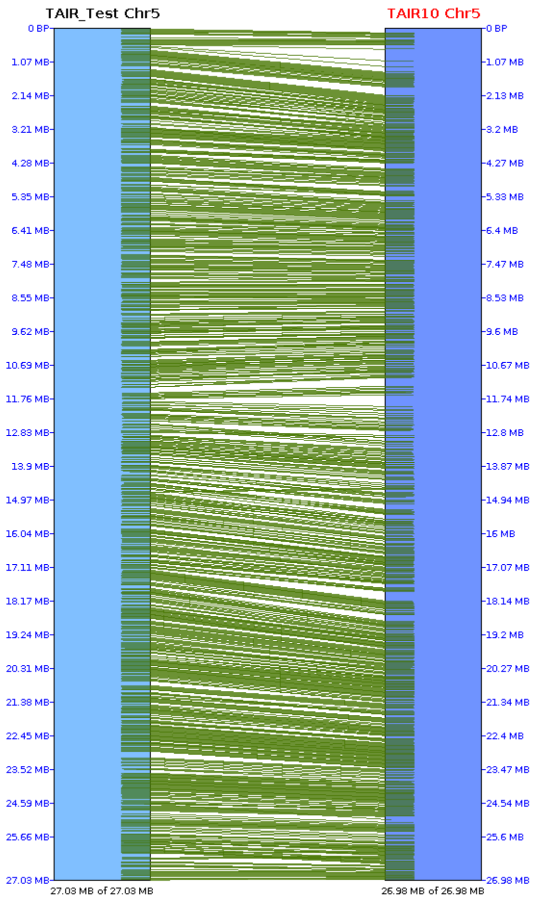

Figure 6. Comparison between original and simulated sequences of each chromosome.

D. Experiment result

A comparison between assembled chromosomes of rice 93-11 and chromosomes of rice Nipponbare were performed (Figure 7). Though gaps existed, the sequence scaffolds were allocated and connected to chromosomes. Most sequence scaffolds were allocated to the corresponding loci on chromosomes by BESs.

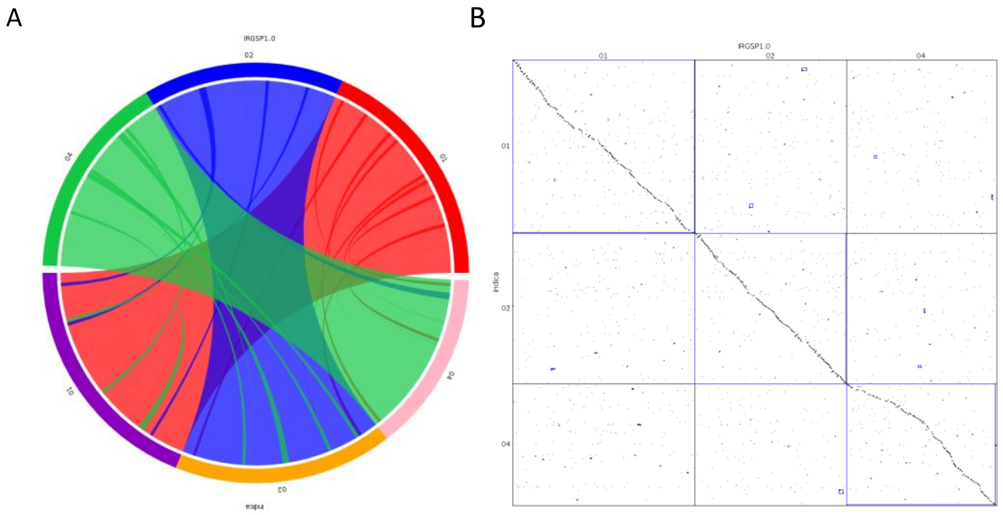

Figure 7. Overall comparison between original and simulated genome sequences. “indica” means the sequences from simulation, and “IRGSP1.0” means the original genome sequence. A. a circle view of alignments. B. a dot-plot view.

Reference

1. Shi, X., Zeng, H., Xue, Y. and Luo, M. (2011) A pair of new BAC and BIBAC vectors that facilitate BAC/BIBAC library construction and intact large genomic DNA insert exchange. *Plant methods*, **7**, 33.
2. Wang, C., Shi, X., Liu, L., Li, H., Ammiraju, J.S., Kudrna, D.A., Xiong, W., Wang, H., Dai, Z., Zheng, Y. *et al.* (2013) Genomic Resources for Gene Discovery, Functional Genome Annotation, and Evolutionary Studies of Maize and Its Close Relatives. *Genetics*, **195**, 723-737.
3. Cock, P.J., Fields, C.J., Goto, N., Heuer, M.L. and Rice, P.M. (2010) The Sanger FASTQ file format for sequences with quality scores, and the Solexa/Illumina FASTQ variants. *Nucleic Acids Res*, **38**, 1767-1771.
4. Soderlund, C., Nelson, W., Shoemaker, A. and Paterson, A. (2006) SyMAP: A system for discovering and viewing syntenic regions of FPC maps. *Genome Res*, **16**, 1159-1168.
5. Soderlund, C., Bomhoff, M. and Nelson, W.M. (2011) SyMAP v3.4: a turnkey synteny system with application to plant genomes. *Nucleic Acids Res*, **39**, e68.
