# Supplementary material for: Whole Genome Mapping with Feature Sets from High-Throughput Sequencing Data
Source: PLoS One. 2016 Sep 9;11(9):e0161583. doi: 10.1371/journal.pone.0161583 (PMC5017645; doi:10.1371/journal.pone.0161583)
Supplement: S5 Table — All considered parameters were listed to help to make the best pooling strategy of whole genome sequencing. (PDF) [file pone.0161583.s010.pdf]

Table S5 Summary of parameters and suggestions.

Parameter	Affected item	Impact trend	Suggestion
Pool dimension	F-set	+	6D is necessary, 9D or larger is better
Pooling strategy	F-set	=	Solid pooling is recommended
Sequencing quality	F-set	+	Higher quality is better
Sequencing depth	F-set	+-	20X is necessary, but should be limited
Prefix sequence length	FS-set	=	5-6 bp is sufficient
Prefix sequence number	FS-set	=	1-4 prefixes is recommended
Filtering frequency	F-set	=	1 is recommended, 2 or larger is required for very large sequencing depth and/or low sequencing quality
FS/k-mer length	F-set	=	Shorter is better for small genomes and longer is better for large genomes; 31 bp is sufficient for any genome
Pool coverage	F-set	-	Smaller is better
Read length	Assembly	+	Longer is better
Sequence library	Integration	=	Different size libraries are better for connecting sequence contigs
BAC library coverage	Integration	+	Greater coverage is beneficial to integrate sequence contigs to physical contigs
BAC clone insert size	F-set	+	Larger is better for large genomes
Genome size	F-set	=	The clone libraries of larger genomes can be split into several groups
Repeat sequences	All steps	=	For genomes with more repeats, smaller pool coverage and larger pool dimensions are preferable

+: An increase in this parameter is beneficial for the affected item

-: An increase in this parameter is unfavorable for the affected item

+-: A balance exists between this parameter and the affected item

=: Please refer to the suggestion
