# Supplementary material for: Whole Genome Mapping with Feature Sets from High-Throughput Sequencing Data
Source: PLoS One. 2016 Sep 9;11(9):e0161583. doi: 10.1371/journal.pone.0161583 (PMC5017645; doi:10.1371/journal.pone.0161583)
Supplement: S2 Data — (ZIP) [file pone.0161583.s003.zip › S2_Data.zip!/S2_Data/index.html]

Result data of Feature Sets-based Genome Mapping


Main result data of simulation for genome of *Arabidopsis thaliana* | | ||  |  |  |
| --- | --- | --- |
| File Name | Description | Link |
| Pool index file | In the pool index file, each row contains a pool name and clone names in this pool.Pool name and clone names were separator by tab character. Clone names were separated by ",". | download |
| Clone feature sequences | It's a zip file contianing all clones' feature sequences. Each feature sequence file end with ".cs" is a binary file. In binary mode, 8 bytes (64 bits) descripts an integer value conresponding an feature sequence. | download |
| Makers | It contains all markers used in the simulation of *Arabidopsis thaliana* genome. | download |
| Physical map | It's a zip file containing all physical map file from the program of FPC(V9.4). The dir of "Sizes" contains the original "sizes" file compatible to FPC. | download |
|  |  |  |

---

Main result data of testing for chr1,2 and 4 of *Oryza sativa indica* 93-11 | | ||  |  |  |
| --- | --- | --- |
| File Name | Description | Link |
| Pool index file | In the pool index file, each row contains a pool name and clone names in this pool.Pool name and clone names were separator by tab character. Clone names were separated by ",". | download |
| Clone feature sequences | It's a zip file contianing all clones' feature sequences. Each feature sequence file end with ".cs" is a binary file. In binary mode, 8 bytes (64 bits) descripts an integer value conresponding an feature sequence. | download |
| Physical map | It's a zip file containing all physical map file from the program of FPC(V9.4). The dir of "Sizes" contains the original "sizes" file compatible to FPC. | download |
|  |  |  |
